# The Identification of *Trans*-acting Factors That Regulate the Expression of *GDF5* via the Osteoarthritis Susceptibility SNP rs143383

**DOI:** 10.1371/journal.pgen.1003557

**Published:** 2013-06-27

**Authors:** Catherine M. Syddall, Louise N. Reynard, David A. Young, John Loughlin

**Affiliations:** Musculoskeletal Research Group, Institute of Cellular Medicine, Newcastle University, Newcastle upon Tyne, United Kingdom; KU Leuven, Belgium

## Abstract

rs143383 is a C to T transition SNP located in the 5′untranslated region (5′UTR) of the growth differentiation factor 5 gene *GDF5*. The T allele of the SNP is associated with increased risk of osteoarthritis (OA) in Europeans and in Asians. This susceptibility is mediated by the T allele producing less *GDF5* transcript relative to the C allele, a phenomenon known as differential allelic expression (DAE). The aim of this study was to identify *trans*-acting factors that bind to rs143383 and which regulate this *GDF5* DAE. Protein binding to the gene was investigated by two experimental approaches: 1) competition and supershift electrophoretic mobility shift assays (EMSAs) and 2) an oligonucleotide pull down assay followed by quantitative mass spectrometry. Binding was then confirmed *in vivo* by chromatin immunoprecipitation (ChIP), and the functional effects of candidate proteins investigated by RNA interference (RNAi) and over expression. Using these approaches the *trans*-acting factors Sp1, Sp3, P15, and DEAF-1 were identified as interacting with the *GDF5* 5′UTR. Knockdown and over expression of the factors demonstrated that Sp1, Sp3, and DEAF-1 are repressors of *GDF5* expression. Depletion of DEAF-1 modulated the DAE of *GDF5* and this differential allelic effect was confirmed following over expression, with the rs143383 T allele being repressed to a significantly greater extent than the rs143383 C allele. In combination, Sp1 and DEAF-1 had the greatest repressive activity. In conclusion, we have identified four *trans*-acting factors that are binding to *GDF5*, three of which are modulating *GDF5* expression via the OA susceptibility locus rs143383.

## Author Summary

GDF5 is an important growth factor that plays a vital role in the development and repair of articulating joints. rs143383 is a polymorphism within the regulatory region of the *GDF5* gene and has two allelic forms, C and T. Genetic studies have demonstrated that the T allele is associated with an increased risk of osteoarthritis in a range of ethnic populations whilst previous functional studies revealed that this allele mediates its effect by producing less *GDF5* transcript than the C allele. In this study, we sought to identify transcription factors that are binding to rs143383 and that are responsible for mediating this differential level of expression. Using two different approaches we have identified four factors and our functional studies have revealed that three of these factors repress *GDF5* expression and that DEAF-1 modulates the differential expression of the two rs143383 alleles. The factors that we have identified could serve as novel therapeutic targets, with their depletion restoring the expression levels of *GDF5* in patients with the osteoarthritis susceptibility T allele. The relevance of our results extends beyond osteoarthritis, since the T allele of rs143383 is also a risk factor for a number of other musculoskeletal diseases.

## Introduction

Osteoarthritis (OA) is a common disease of the synovial joints, affecting millions of people worldwide. It is a chronic, highly disabling disease, characterised by the progressive loss of articular cartilage, changes in the subchondral bone, and variable levels of synovial inflammation [1]. Many patients suffer from joint pain and tenderness, limiting the functioning of the joint and thus having a significant impact on quality of life. Furthermore, evidence is now emerging of an increased mortality risk in OA patients [2].

Non-steroidal anti-inflammatory drugs (NSAIDs) and cyclo-oxygenase 2 (COX-2) inhibitors are recommended for the pharmacological management of OA. Although these have proven to be effective for pain relief and suppression of inflammation, these treatments are failing to target the underlying cause and progression of disease. There has been limited success so far in trials of disease-modifying drugs, with arthroplasty remaining the basis for curative therapy [3].

There are a number of risk factors for OA, including age, gender, mechanical injury and obesity. Genetics contribute a significant risk to developing the disease, with heritability estimates ranging from 39–79% dependent on the joint site affected [4]. A number of genes have been found to harbour OA susceptibility alleles and genome wide association scans have provided additional loci worthy of investigation [5]. When a susceptibility allele has been identified it is necessary to investigate the functional effect of the polymorphism in order to enhance understanding of its role in disease aetiology. This information can then be used to assist in diagnosis, prognosis and to alleviate detrimental genetic effects by modulating or restoring gene function or expression.

To date, the most reproducible association with OA has been to rs143383, a C/T single nucleotide polymorphism (SNP) located within the 5′untranslated region (5′UTR) of the growth differentiation factor 5 gene *GDF5* (HUGO Gene Nomenclature Committee (HGNC) number 4420). The T allele of the SNP was first associated with increased risk of OA in an Asian population, with this association subsequently replicated in Europeans [6]–[8]. Haplotype analysis combined with an examination of promoter activity following the sequential deletion of the *GDF5* promoter/5′UTR demonstrated that rs143383 is the causal SNP, with its T allele mediating reduced expression relative to its C allele [6]. This phenomenon is known as differential allelic expression (DAE). A subsequent analysis of RNA extracted from the joint tissues of OA patients heterozygous for the SNP revealed that the *GDF5* DAE is active during the disease process, with DAE observed in cartilage, ligament, synovium, fat pad and meniscus [7], [9]. Overall, these studies demonstrated that a reduction in *GDF5* expression mediated by the T allele of rs143383 is a risk factor for OA.

GDF5 protein has a vital role in the formation and repair of joints. It acts as an extracellular signalling molecule, activating the expression of genes involved in the formation of cartilage and bone [10]. During joint specification, GDF5 is present within the joint interzone, and has been found to have a pivotal role during chondrogenesis [11]. It is expressed in both normal and OA cartilage, and has been proposed to also be important in cartilage repair following trauma [12]–[16]. Rare and highly penetrant mutations of *GDF5* underlie several severe musculoskeletal conditions, including Hunter-Thompson syndrome, Grebe syndrome and Brachdactyly Type C [17]–[20]. These conditions present with joint dislocations, which are found to mainly occur in the knees and hips, shortened limb bones, abnormalities in the development of the phalangeal joints and brachydactyly.

This essential role of GDF5 during joint development and joint maintenance has been further demonstrated in the mouse brachypodism mutation, which is a premature termination codon of *Gdf5* that results in an absence of functional protein from the mutant allele. Homozygous mice have a number of developmental abnormalities of both bone and soft tissues whereas heterozygous mice show no overt growth abnormalities but when challenged are more susceptible to develop an OA-like phenotype [21], [22].

We have previously reported on DEAF-1 (HGNC:14677) as a potential *trans*-acting factor that binds to rs143383 [9]. The aim of our latest study was to perform a more detailed analysis of DEAF-1 and to identify additional factors that bind differentially to the two alleles of rs143383 and that could account for the *GDF5* DAE that is mediated by this SNP. We used the human liposarcoma cell line SW872 for our research since 1) the cell line expresses *GDF5*; 2) it is heterozygous for rs143383; 3) it also demonstrates *GDF5* DAE and 4) it is amenable to a variety of *in vitro* experimental manipulations. Since SW872 cells exhibit *GDF5* DAE it was assumed that the *trans*-acting factors that mediate the DAE were expressed in these cells.

We used two different approaches to identify the novel *trans*-acting factors. The first utilised bioinformatics software to predict protein binding based on the sequence surrounding rs143383, followed by electrophoretic mobility shift assays (EMSAs) to screen these potential candidates. The second approach used an oligonucleotide pull down assay to isolate proteins binding to the promoter region of *GDF5*, followed by quantitative mass spectrometry, enabling both the identification and quantification of proteins binding to the C and T alleles of rs143383. Chromatin Immunoprecipitation (ChIP), luciferase assays and RNA interference (RNAi) were then used to confirm binding of the newly identified candidate proteins *in vivo* and to assess their role in mediating *GDF5* DAE. The EMSA and RNAi results were then confirmed using a combination of the chondrosarcoma cell line SW1353, the osteosarcoma cell line MG63 and human articular chondrocytes. This study has identified four *trans*-acting factors that are binding to *GDF5*, three of which are modulating the expression of this important growth factor.

## Results

### SW872 is a suitable cell line to investigate the*GDF5* DAE mediated by rs143383

**Figure 1:**
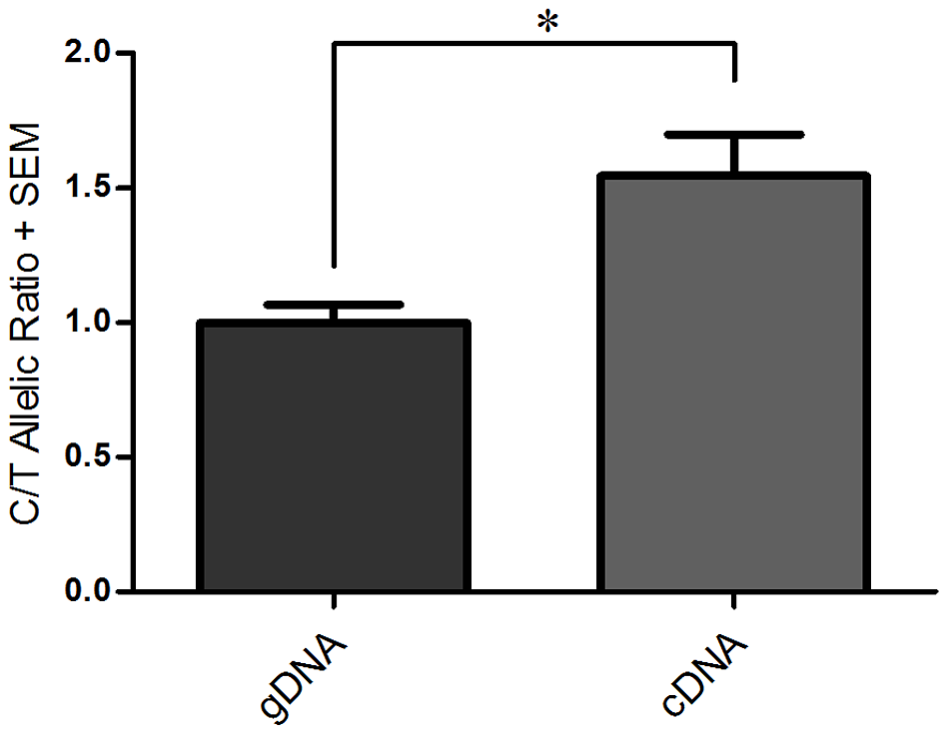
Differential allelic expression (DAE) of*GDF5* in SW872 cells assessed using rs143383. The C/T allelic ratio for genomic DNA (gDNA) and complementary DNA (cDNA) are shown. Genomic DNA was normalised to 1.0 and then used to compare against the C/T allelic ratio obtained for cDNA. Error bars denote the standard error of the mean (SEM). * p<0.05, calculated using a Students 2 tailed *t*-test, n number of 3.

As we previously described, the human liposarcoma cell line SW872 is heterozygous at rs143383, expresses *GDF5* and demonstrates DAE [23]. In this cell line there is a DAE imbalance of 1.5 between the C and T alleles (Figure 1), which is comparable to the average DAE observed in human joint tissues [9]. In that study the level of DAE at rs143383 was found to be similar between all the joint tissues examined, and was confirmed in several different cell lines using luciferase reporter assays [9]. This indicates that the imbalance is not due to a tissue or cell type specific factor, but instead implies that the same *trans*-acting factors are regulating the expression of *GDF5* via rs143383 in a number of cell types. We therefore used the SW872 heterozygous cell line as a model system for the discovery and investigation of these *trans*-acting factors.

### Initial assessment of*trans*-acting factor binding to rs143383 by EMSAs

**Figure 2:**
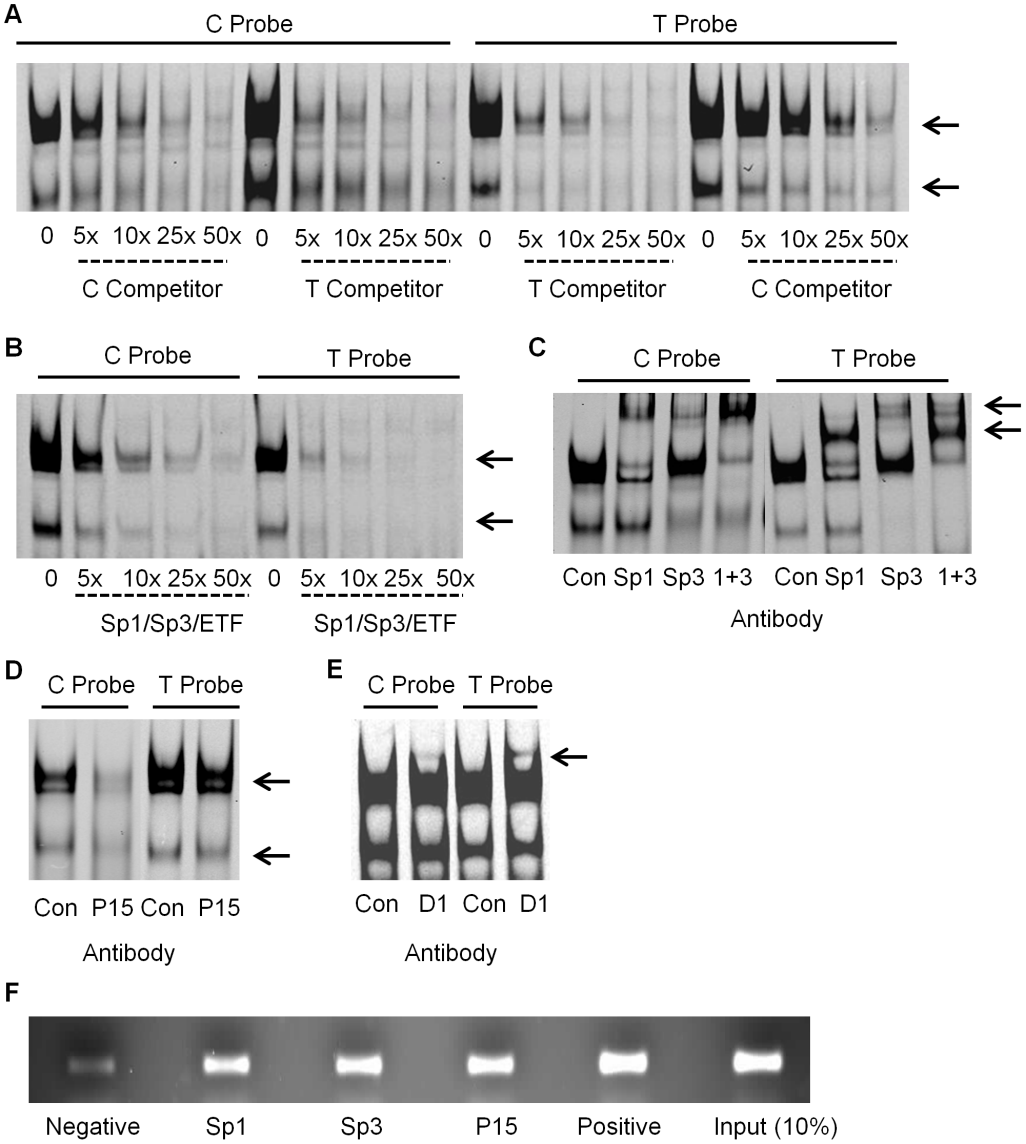
EMSA and ChIP analysis in SW872 cells. (**A**) The addition of increasing concentrations of unlabelled C and T allele competitor were added to the EMSA reaction containing the C and the T allele probes and SW872 nuclear extract, with the arrows indicating the specific complexes binding to the probes. (**B**) The addition of increasing concentrations of the Sp1/Sp3/ETF unlabelled consensus competitor to the EMSA reaction containing the C or T allele probe. The arrows indicate the two complexes that are competed. (**C**) Supershift experiment demonstrating the effect of adding antibodies targeting Sp1, Sp3, and Sp1 and Sp3 together (1+3), compared to the IgG rabbit antibody control (Con) to the EMSA reaction containing the C or T allele probe. The arrows indicate the supershifted complexes. (**D**) Demonstration of the effect of adding P15 antibody to the EMSA reaction, compared to the IgG rabbit antibody control (Con). The arrows indicate the affected complexes. (**E**) Demonstration of the effect of adding DEAF-1 (D1) antibody to the EMSA reaction, compared to the IgG rabbit antibody control (Con). The arrow indicates a supershifted complex. (**F**) ChIP analysis of Sp1, Sp3 and P15. Sheared genomic DNA was immunoprecipitated with Sp1, Sp3, P15, rabbit polyclonal IgG (negative control) and anti-acetyl histone H3 (positive control) antibodies and then PCR amplified across exon 1 of *GDF5*. The input represents 10% of the non-immunoprecipitated sheared genomic DNA.

We investigated protein complex binding using SW872 nuclear extract and fluorescently labelled C and T allele probes (Figure 2A). We observed a similar pattern of protein complex binding to the two probes. We confirmed the specificity of the assay by adding unlabelled C and T allele competitors, and the two specific complexes binding revealed a differential affinity for the two alleles. For both complexes, binding to the C allele probe was outcompeted with excess unlabelled C and T allele competitor, and *vice versa* for the T allele probe. Higher concentrations of C allele unlabelled competitor were required to outcompete binding to the T allele probe and complex binding was competed from the C allele probe at a lower concentration of T allele competitor compared to C allele competitor. These results suggest the two protein complexes bind more avidly to the T allele, compared to the C allele. We used smaller sized unlabelled competitors to refine the region of binding of the two complexes; this assay suggested that the majority of the sequence of the probe including the rs143383 polymorphic site is required for the binding of the two complexes (Figure S1). There is a small degree of competition using the 50× concentration of competitor 1 (−15 to +2 relative to rs143383) and competitor 2 (−6 to +6) but not with competitor 3 (−3 to +10) suggesting that the region upstream of the polymorphism may be more important for complex 1 and 2 binding.

### Identification of the binding of Sp1 and Sp3

Using the online databases TransFac, Tess and Promo 3.0, we identified a number of transcription factors that were predicted to bind to *GDF5* within the region containing rs143383. We refined the number of potential factors using competitors containing the consensus binding sequence of each factor (competitor sequences are listed in Table S1). If binding of either complex to the *GDF5* probes was competed, the factors were investigated further by the addition of an antibody targeting the protein to the EMSA binding reaction. On the addition of a shared Sp1/Sp3/ETF consensus competitor, binding of both complexes to the *GDF5* probes was competed (Figure 2B). Sp1 (HGNC:11205) and Sp3 (HGNC:11208) had been identified by all three databases. The addition of an antibody targeting Sp1 resulted in a supershift of the upper complex and addition of an antibody targeting Sp3 supershifted both the lower, and one of the upper complexes (Figure 2C). The Sp1 and Sp3 antibodies were the only ones tested that resulted in supershifts; Figure S2 shows examples of *trans*-acting factors that did not supershift, along with a supershifted Sp1. These results confirm the binding of Sp1 and Sp3 to *GDF5 in vitro* in SW872 cells. We subsequently confirmed the binding of Sp1 and Sp3 using nuclear extracts from the chondrosarcoma cell line SW1353, the osteosarcoma cell line MG63 and from primary human articular chondrocytes (HACs; Figure S3A and S3B).

### Identification of the binding of P15

We performed an oligonucleotide pull down assay using C and T allele DNA probes and then identified and quantified the binding of proteins to each allele using tandem mass tag (TMT) 6-plex isobaric labelling followed by mass spectrometry. The binding of activated RNA polymerase II transcriptional coactivator p15 (P15; also known as SUB1 and PC4; HGNC:19985) was identified in both the C and T allele DNA samples. However, P15 was reproducibly found to be more abundant in the T allele sample, in comparison with the C allele sample with an average C/T ratio of 0.67. This protein was absent in the background control sample. P15 does not have a known binding consensus sequence and we were therefore not able to use an EMSA to investigate competition for binding to the fluorescently labelled C and T allele probes. However, on the addition of an antibody targeting P15, we observed a decrease in the two specific protein complexes binding to the two probes (Figure 2D). This was also observed in SW1353 and MG63 cells and in HACs (Figure S3C).

### Demonstration of the binding of DEAF-1

Following our previous report that the DEAF-1 consensus competitor sequence was able to compete binding of proteins to C and T allele probes [9], we investigated the effect of adding an antibody targeted against DEAF-1 to our EMSA reaction. We observed a supershifted complex in both C and T allele probe reactions, with the complex appearing to be more intense in the T allele probe sample (Figure 2E). The supershifted complex was also confirmed using nuclear extract from HACs, with the protein complexes binding to the C and T allele probes being less intense than those observed in the SW872 cells (Figure S3D).

P15 was discovered by the oligonucleotide pull down experiment but this technique did not detect Sp1, Sp3 or DEAF-1, which were instead detected by the EMSA analysis. A possible explanation for this is the different binding conditions used, including different salt concentrations, in the pull down assay versus EMSA. To assess this, we repeated the EMSA using salt concentrations equivalent to those used in the pull down and observed that Sp1 and Sp3 were no longer able to bind to the C and T allele probes (Figure S4). We suspect therefore that this accounts for the different results obtained between pull down and EMSA. This result justifies our use of two distinct techniques for identifying *trans*-acting factors.

### Sp1, Sp3, and P15 bind to*GDF5 in vivo*

Following the identification and confirmation of the binding of the Sp1, Sp3, P15 and DEAF-1 *trans*-acting factors to a *GDF5* probe *in vitro*, we next sought to confirm the binding of these factors to the *GDF5* locus *in vivo* using ChIP followed by PCR. In the PCR reaction we amplified the *GDF5* exon 1 region, encompassing rs143383, and the intensities of the PCR products were clearly greater following ChIP with anti-Sp1, anti-Sp3 and anti-P15 antibodies relative to the IgG negative control antibody (Figure 2F). This suggests that this region of *GDF5* is enriched for Sp1, Sp3 and P15 binding. We were unable to examine binding of DEAF-1 *in vivo* due to the unavailability of a specific ChIP grade antibody for this protein.

### Sp1, Sp3, P15, and DEAF-1 regulate*GDF5* transcriptional activity

**Figure 3:**
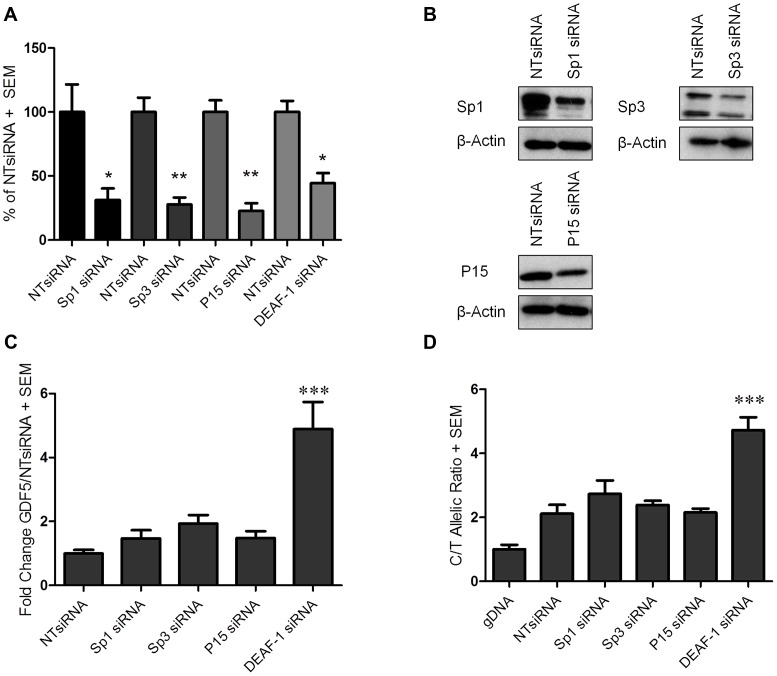
*GDF5* expression following Sp1, Sp3, P15 and DEAF-1 depletion. (**A**) Expression levels of *Sp1*, *Sp3*, *P15* and *DEAF-1* mRNA are shown as a percentage of the control non-targeting siRNA (NTsiRNA) treated cells following *Sp1*, *Sp3*, *P15* and *DEAF-1* siRNA knockdown. Error bars denote the standard error of the mean (SEM). *p<0.05, calculated relative to the NTsiRNA value using a Students 2 tailed *t*-test. (**B**) Immunoblots demonstrating Sp1, Sp3 and P15 protein depletion following siRNA treatment. Protein extracted from cells treated with the NTsiRNA control were used for basal protein expression whilst β-Actin was used as a loading control. (**C**) Fold change in *GDF5* expression following *Sp1*, *Sp3*, *P15* and *DEAF-1* siRNA knockdown and shown relative to the NTsiRNA control. Error bars denote the SEM. ***p<0.001, calculated using a ANOVA. (**D**) The rs143383 C/T allelic ratio is shown following *Sp1*, *Sp3*, *P15* and *DEAF-1* siRNA knockdown and compared against treatment with the NTsiRNA control. Allelic ratios were normalised to genomic DNA (gDNA). Error bars denote the SEM. ***p<0.001, calculated using a ANOVA. Each siRNA experiment was performed 3 times each with an n of 3.

After confirming the binding of these four factors to *GDF5*, we then sought to assess if each factor regulates the expression of *GDF5*. We first confirmed the expression of *Sp1*, *Sp3*, *P15* and *DEAF-1* in patient tissue samples; all four genes, in addition to *GDF5*, were expressed in cartilage (from OA and non-OA patients), synovium and fat pad (Figure S5). We next analysed the effect of Sp1, Sp3, P15 and DEAF-1 depletion on *GDF5* expression by RNAi in the SW872 cells. The depletion of the mRNA for each gene was confirmed by real time RT-PCR and of Sp1, Sp3 and P15 protein by immunoblotting (Figures 3A and 3B). Due to the low expression levels of DEAF-1 within SW872 cells, we had difficulty in confirming the knockdown of the endogenous protein. We therefore confirmed that the siRNA is able to deplete DEAF-1 protein following the over expression of DEAF-1 EGFP fusion protein (Figure S6).

The overall expression of *GDF5* was increased following depletion of each factor. For Sp1, Sp3 and P15 depletion, these increases in *GDF5* expression were not significant, whilst a significant fold change (p<0.001) was observed upon DEAF-1 knockdown (Figure 3C). We next used allele specific real time PCR to assess if any of the four factors differentially affects expression of the two alleles of rs143383, and as such could contribute to the DAE mediated by this SNP. Depletion of Sp1 and Sp3 resulted in small and non-significant increases in the C to T ratio (ratio of 2.1 in the control (NTsiRNA) to 2.7 (Sp1 siRNA) or 2.4 (Sp3 siRNA)) whilst P15 depletion did not alter the DAE (Figure 3D). DEAF-1 depletion increased the DAE from a C/T ratio of 2.1 in the control (NTsiRNA) to 4.7 (DEAF-1 siRNA) and this was highly significant (p<0.001, Figure 3D).

We confirmed the effect on overall *GDF5* expression in SW1353 cells, with knockdown of the four factors increasing *GDF5* expression. In line with that observed in SW872 cells, the increases in *GDF5* expression were not significant following Sp1, Sp3 and P15 depletion but a significant fold change was observed upon DEAF-1 knockdown in this chondrosarcoma cell line (Figure S7). Additionally Sp1, Sp3, P15 and DEAF-1 depletion experiments were performed in HACs. The depletion of the mRNA for each gene was confirmed by real time RT-PCR and of Sp1, Sp3 and P15 protein by immunoblotting (Figure S8; as for SW872, endogenous DEAF-1 was not detectable in HACs). Depletion of P15 and DEAF-1 resulted in small and non-significant increases in *GDF5* expression, whilst Sp3 depletion increased *GDF5* expression significantly (p<0.05).

These data suggest that all four factors are involved in the transcriptional activity of *GDF5*, each repressing *GDF5* expression, with DEAF-1 having significant repressive effects and also clearly contributing to *GDF5* DAE in the SW872 cells.

### Over expression of Sp1, Sp3, and DEAF-1 represses the C and T alleles

**Figure 4:**
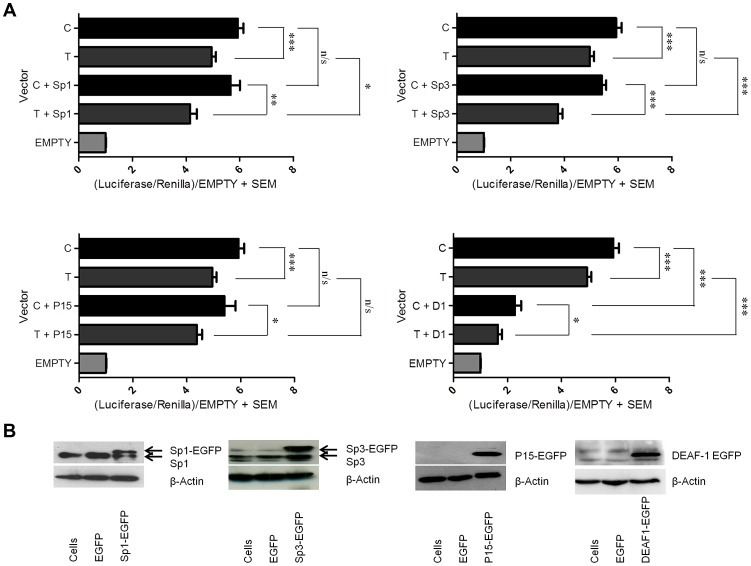
Over expression of the Sp1, Sp3, P15, and DEAF-1 proteins. (**A**) Promoter activity of the C and T *GDF5* luciferase vectors is shown relative to *Renilla*. Values are normalised to the luciferase levels of the EGFP/pGL3 empty vector (EMPTY). Promoter luciferase levels of both C and T allele vectors are shown in addition to the empty EGFP vector (C and T) and following over expression of Sp1 (C+Sp1 and T+Sp1), Sp3 (C+Sp3 and T+Sp3), P15 (C+P15 and T+P15) and DEAF-1 (C+D1 and T+D1). Error bars denote the standard error of the mean (SEM). *p<0.05 **p<0.01 ***p<0.001 n/s = not significant, calculated using a Students 2 tailed *t*-test. Six replicate experiments were performed, each with an n of 4. (**B**) Immunoblots showing Sp1 (Sp1-EGFP), Sp3 (Sp3-EGFP), P15 (P15-EGFP) and DEAF-1 (DEAF1-EGFP) protein levels following over expression compared to the EGFP/pGL3 combination empty vector control (EGFP). Cells are untreated protein samples whilst β-Actin was used as a loading control. The arrows indicate basal protein and over expressed protein levels.

We next over expressed each of the four factors in combination with a reporter vector that contained the *GDF5* promoter and the 5′UTR sequence encompassing rs143383 and which drove expression of the luciferase gene. We used two constructs, one containing the T allele and the other the C allele of the SNP. These experiments were performed in the chondrosarcoma cell line SW1353. We first assessed what effect this single nucleotide difference mediated on luciferase activity and observed that the presence of a T allele at rs143383 significantly reduced the luciferase activity, with an average C/T allelic ratio of 1.2 (p<0.001, Figure 4A), confirming previous findings [9]. Over expression of Sp1, Sp3, P15 and DEAF-1 fusion proteins was then confirmed by immunoblotting and immunofluorescence (Figure 4B and Figure S9 respectively). Over expression of Sp1 decreased the promoter activity of both C and T allele constructs, with a significant repressive effect on the T allele (p<0.05; Figure 4A), significantly increasing the C/T ratio to 1.38 (p<0.01). Over expression of Sp3 decreased the promoter activity of both the C and T allele constructs, and this effect was significant with the T allele construct (p<0.001; Figure 4A) significantly increasing the allelic ratio to 1.48 (p<0.001). P15 over expression decreased the promoter activity of both alleles, however, this repressive effect was not significant (Figure 4A). Finally, DEAF-1 over expression significantly repressed the promoter activity of both alleles (C and T alleles p<0.001; Figure 4A), but most notably repressed the T allele construct, decreasing its activity to near that of the empty control and significantly increasing the allelic ratio to 1.37 (p<0.01). These results confirm that Sp1, Sp3 and DEAF-1 are significantly repressing *GDF5* expression, and this repression is greater for the T allele of rs143383. Conversely, P15 only appears to be mediating a minor, non-significant repressive effect.

### Over expression of Sp1, Sp3, and DEAF-1 in different combinations leads to stronger repressive effects

**Figure 5:**
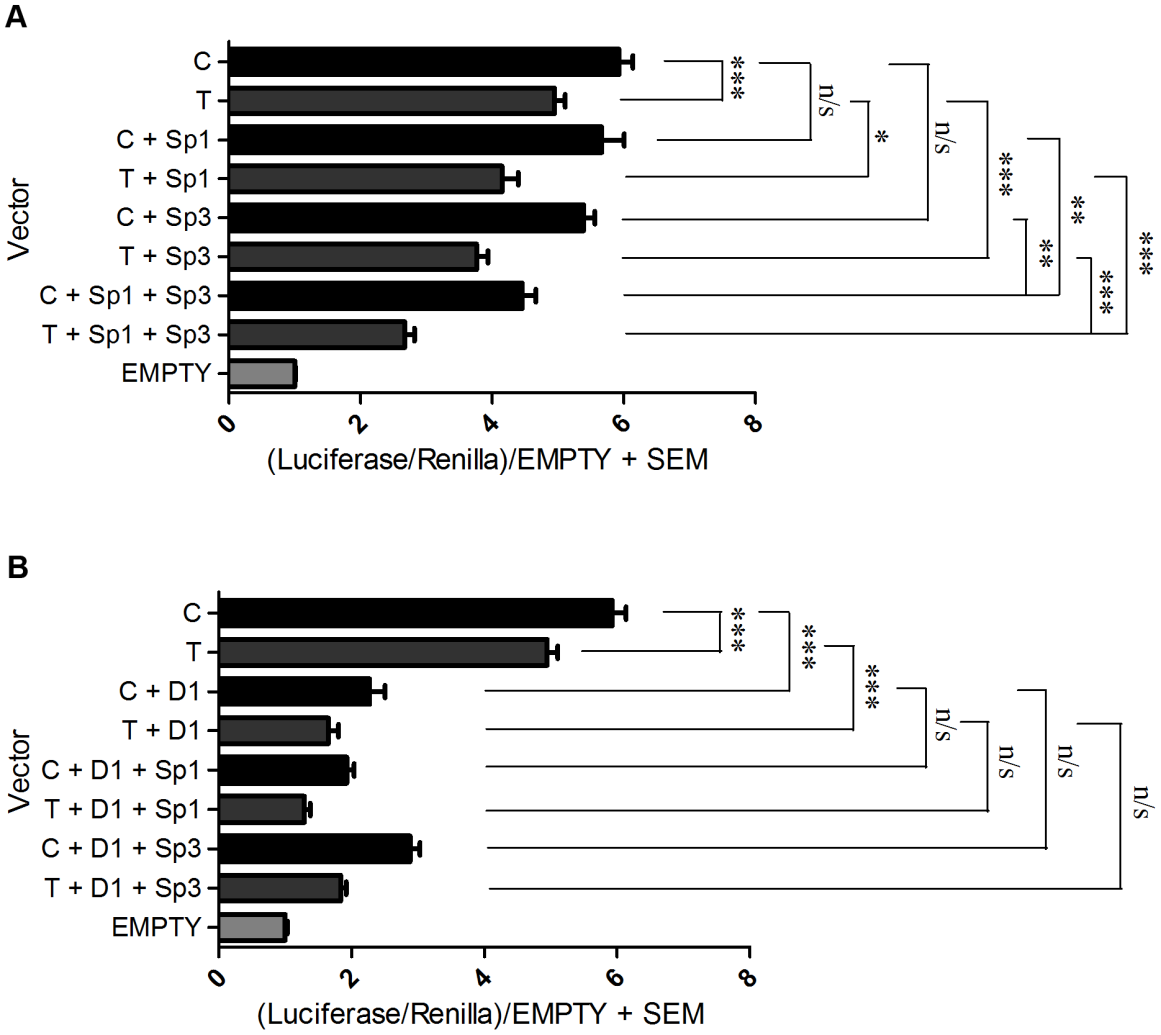
Over expression combinations. Promoter activity of the C and T *GDF5* luciferase vectors is shown relative to *Renilla*. Values are normalised to the luciferase levels of the EGFP/pGL3 empty vector (EMPTY). (**A**) Promoter luciferase levels of both C and T allele vectors are shown in addition to the empty EGFP vector (C and T) and following over expression of Sp1 alone (C+Sp1 and T+Sp1), Sp3 alone (C+Sp3 and T+Sp3) and Sp1 and Sp3 in combination (C+Sp1+Sp3 and T+Sp1+Sp3). (**B**) Promoter luciferase levels of both C and T allele vectors are shown in addition to the empty EGFP vector (C and T) and following over expression of DEAF-1 alone (C+D1 and T+D1), and DEAF-1 in combination with Sp1 (C+D1+Sp1 and T+D1+Sp1) and in combination with Sp3 (C+D1+Sp3 and T+D1+Sp3). Error bars denote the standard error of the mean (SEM). *p<0.05 ***p<0.001 n/s = not significant, calculated using a Students 2 tailed *t*-test. 3 replicate experiments were performed, each with an n of 4.

We next assessed whether the repressive effects seen in the above experiment would be stronger if the factors were co-transfected and over expressed together. When Sp1 and Sp3 were jointly over expressed there was a significantly greater reduction in expression of both the C and the T alleles relative to when they were over expressed alone (Figure 5A). Furthermore, the C/T allelic ratios significantly increased from 1.38 for the Sp1 over expression and 1.48 for the Sp3 over expression to 1.70 for the joint over expression (p<0.001 for the joint over expression versus Sp1 alone and p<0.05 for the joint over expression versus Sp3 alone; Table S2). When Sp1 and DEAF-1 were jointly over expressed there was a reduction in expression of both the C and T alleles relative to when they were over expressed alone (Figure 5B). The C/T allelic ratios increased significantly from 1.38 for Sp1 and 1.37 for DEAF-1 to 1.55 for the joint over expression (p<0.001 versus C/T). However, these C/T allelic ratio changes were not significant when compared with Sp1 or DEAF-1 over expression alone (p = 0.1). Finally, when Sp3 and DEAF-1 were jointly over expressed, the C/T allelic ratios increased from 1.48 for Sp3 and 1.37 for DEAF-1 to 1.6 for the joint over expression, and this was a significant C/T difference compared to DEAF-1 over expression alone (p = 0.01). Over expression of P15 in combination with Sp1, Sp3 or DEAF-1 did not contribute any further significant repressive effects compared to over expression of the factors alone (data not shown).

Finally, we performed co-immunoprecipitation experiments using nuclear extracts from SW1353 cells to show that Sp1, Sp3, P15 and DEAF-1 directly interact. We observed co-immunoprecipitation of Sp1 when Sp3 and DEAF-1-EGFP were immunoprecipitated (Figure S10A). In the reciprocal experiment, Sp3 and DEAF-1 were co-immunoprecipitated upon Sp1 immunoprecipitation (Figure S10B and S10D). P15 was co-immunoprecipitated following Sp1, Sp3 and DEAF-1 EGFP immunoprecipitation (Figure S10C). Finally, Sp3 was co-immunoprecipitated following DEAF-1 EGFP immunoprecipitation (Figure S10B), and the reciprocal experiment revealed DEAF-1 co-immunoprecipitation with Sp3 immunoprecipitation (Figure S10D).

## Discussion

The rs143383 T allele has been reproducibly associated with increased risk of OA, and produces a lower level of expression of *GDF5* relative to the C allele. This DAE is apparent in all tissues of the articulating joint and also within the rs143383 heterozygote cell line SW872, which therefore provided us with an ideal model system to investigate the *trans*-acting factors mediating this DAE [9], [23].

Using a variety of techniques we identified Sp1, Sp3, P15 and DEAF-1 as proteins that bind to the two alleles of rs143383. Depletion of all four increased the expression of *GDF5*, whilst DEAF-1 depletion significantly modulated the DAE. Conversely, the over expression of Sp1, Sp3 and DEAF-1 repressed C and T allele expression, repressing the T allele more strongly. When over expressed together, DEAF-1 and Sp1 mediated the greatest overall repressive effect whereas over expression of Sp1 and Sp3 together mediated the greatest differential allelic effect, repressing the T allele to a greater extent than the C allele. Using co-immunoprecipitation we demonstrated that these four factors directly interact with each other.

Overall therefore we have identified *trans*-acting factors that bind differentially to the alleles of rs143383 and which contribute to the DAE that is mediated by this important OA susceptibility locus.

Sp1 and Sp3 are well characterized transcription factors that have a high degree of conservation between their zinc finger DNA binding domains (95% homology) and which bind to related DNA sequences [24], [25]. Sp1 is usually considered a potent activator of gene expression, although repressive activity has been reported, whereas Sp3 is known to possess both activator and repressor functions [26]–[28]. Both proteins are ubiquitously expressed and bind with high affinity to GC rich motifs, which are promoter elements present in a diverse range of genes. The proteins also form a multi-protein complex to synergistically regulate gene expression [29]. Promoters that do not contain a TATA binding site are commonly known to have an Sp protein-binding site. In these TATA-less promoters Sp1 has been reported to play a critical role in anchoring the basal transcription machinery to promote transcriptional initiation. Sp1 facilitates the binding of TFIID through binding to TBP (TATA binding protein) associated factors (TAFs) which then recruit RNA polymerase II [30]. *GDF5* does not contain a TATA box and thus it appears likely that in binding to the *GDF5* 5′UTR, Sp1 and Sp3 may be mediating interactions with the basal transcriptional machinery to modulate transcription of this gene.

In our EMSA experiments, a comparison of the complex formation of Sp1 and DEAF-1 to the *GDF5* probes revealed that there is an abundance of Sp1 protein relative to DEAF-1 protein. DEAF-1 however has the most significant repressive effect on *GDF5* expression. Sp1 is known to form homomultimers when it is bound to the promoters of genes, where it can serve as a docking site for the binding of other proteins [31]. This Sp1 multimerisation may account for the relative abundance of this protein.

Sp1 and Sp3 have been previously reported to interact with HDAC1 in order to mediate gene repression [32]. Our analysis did not however provide evidence of HDAC1 binding to rs143383 or to its immediate flanking sequence. The importance of Sp1 and Sp3 during joint development is highlighted by the large number of target genes that they regulate, the expression of which are key for the formation of the joint and include *SOX9*, *COL1A1* and *RUNX2* [33]–[36].

P15 is a small, highly abundant nuclear protein with multiple functions in transcription, replication and DNA repair [37]. As a transcriptional co-activator, P15 mediates functional interactions between transcription factors and the general transcription machinery [38]. P15 has also been reported to stabilize multi-protein complexes and has previously been reported to act as a co-activator of Sp1, where it was reported to function as a linker between Sp1 and the pre-initiation complex (PIC) [39], [40]. Repressor functions of P15 have also been reported [41]. P15 knockout mice are lethal, highlighting the important role of this factor during development; however heterozygous knockout mice display no overt phenotype indicating there may be a threshold level of P15 that is required for normal development.

Sp1, Sp3 and DEAF-1 were not identified by the oligonucleotide pull down experiment. We hypothesised that this may be due to the different salt conditions used between pull down and EMSA and we then demonstrated that this was the case. This highlights the importance of using more than one method for the discovery of *trans*-acting factors. Another difference between our pull down and EMSA experiments was the length of the genomic DNA sequence used, which was 212 bp in the pull down and 33 bp in the EMSAs. We chose to use a long sequence in the pull down in order not to limit the capture of proteins that may bind over large DNA regions. It is possible however that by using such a long sequence we captured non-specific proteins that may have disrupted the binding of Sp1, Sp3 and DEAF-1. The use of a shorter DNA sequence or of repeat concatamers of rs143383 and its immediate flanking sequence, combined with varying salt concentrations, may have led to the identification of Sp1, Sp3 and DEAF-1 by the oligonucleotide pull down approach.

Of all of the four *trans*-acting factors that we identified, DEAF-1 appears to repress *GDF5* expression most significantly. The lack of a ChIP grade antibody precluded us from demonstrating the binding of DEAF-1 *in vivo*. However, the EMSA supershift that we observed combined with the significant changes in both overall and allelic *GDF5* expression following DEAF-1 depletion, and the significant repressive effects observed following DEAF-1 over expression, provided us with compelling evidence that this *trans*-acting factor is modulating *GDF5* expression at rs143383. DEAF-1 is repressing the T allele more avidly, compared with the C allele, thus following DEAF-1 depletion we expected to observe a greater increase in the expression of the T allele, and a decrease in the C/T allelic ratio. Conversely, we observed an increase in the C/T allelic ratio. We believe this may be either a result of the incomplete depletion of DEAF-1 protein, or because the other factors forming part of the repressive complex are continuing to differentially repress *GDF5* expression.

DEAF-1 is expressed in many neuroendocrine and reproductive tissues and is expressed at high levels in the foetus, suggesting an important role during development [42]. DEAF-1 regulates the expression of a number of genes and its transcriptional activity can be modulated by a single base-pair change to its binding site, with its repressive regulation of the expression of the serotonin auto-receptor 1A (*5HT1A*) gene reduced following a C to G transversion [43], [44]. This study confirms our observation that the activity of DEAF-1 is sensitive to subtle changes in its binding sequence. DEAF-1 knockout mice display skeletal abnormalities including rib cage defects, with a large proportion of the animals suffering from defective neural tube closure that causes death shortly after birth [45].

**Figure 6:**
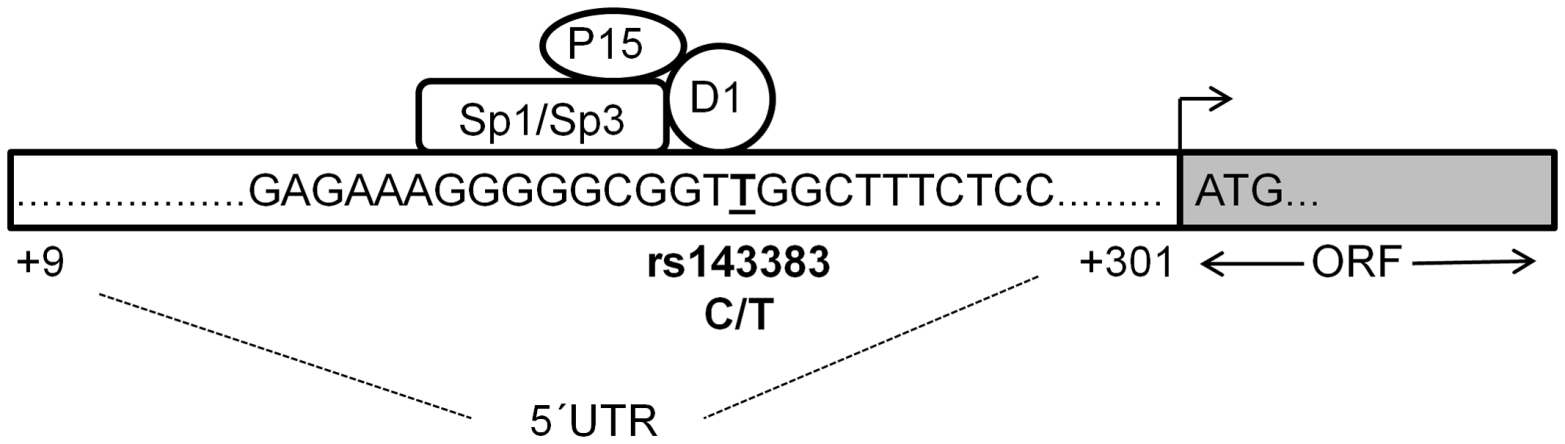
Proposed binding model of the four*trans*-acting factors to rs143383. A region (+9 to +301 relative to the transcription start site) of the *GDF5* 5′UTR is depicted, with the sequence immediately flanking rs143383 (T allele underlined) shown. We propose that DEAF-1 binds directly to rs143383 (at the TTGG site) and that Sp1 and Sp3 bind just upstream (to the Sp site GGGCGG), mediating a repressive effect through DEAF-1. P15 may be interacting with the repressive multi-protein complex and serving as a linker with the general transcription machinery. ORF is the open reading frame of *GDF5* whilst ATG is the translation initiation codon.

Using our experimental data and the predicted binding regions for each protein we have prepared a model for how we believe Sp1, Sp3, P15 and DEAF-1 are interacting relative to rs143383 (Figure 6). The core consensus site for DEAF-1 is TCGG, which resides directly over the SNP, whereas the Sp1/Sp3 GC binding motif is immediately upstream. Although we have confirmed the binding of P15 to *GDF5* both *in vitro* and *in vivo*, P15 is not mediating a significant repressive effect on *GDF5* expression. We propose therefore that DEAF-1, Sp1 and Sp3 are forming a repressive complex that forms directly over rs143383 and are differentially modulating the expression of the C and T alleles. P15 may be interacting with this complex and serving as a linker between Sp1 and the general transcription machinery. We have very recently identified YY1 as a transcriptional activator that binds 80 bp upstream of rs143383, within the *GDF5* promoter [46]; YY1 and Sp1 have previously been shown to jointly modulate the expression of genes and so it is possible that YY1 may indirectly interact with the complex at rs143383 [47].

The relevance of our results extend beyond OA, since the T allele of rs143383 has been associated with a number of other musculoskeletal phenotypes including congenital hip dysplasia [48], Achilles tendinopathy [49], lumbar disc degeneration [50], variation in normal height, hip axis length, and an increased risk of fracture [51], [52]. Transcription factors are now becoming more widely considered as targets for therapeutics to modulate the expression of genes. One approach that has proven effective *in vivo* and which is being considered for clinical application is the inhibition of transcription factors with molecules that mimic the transcription factor binding site [53]. This is known as transcription factor decoy and Sp1 has already been targeted using this approach in breast cancer [54]. The factors that we have identified could therefore serve as novel therapeutic targets, with their depletion restoring the expression levels of *GDF5* in patients with the OA susceptibility T allele.

## Materials and Methods

### Cell culture

SW872 cells were cultured in Dulbecco's modified eagles medium: Hams F12 nutrient mix, GlutaMAX in a 3∶1 ratio (Invitrogen, Life Technologies, Paisley, UK) containing 5% (v/v) foetal bovine serum (FBS), 100 U/ml penicillin and 100 µg/ml streptomycin (Sigma-Aldrich, St. Louis, USA). SW1353 cells were cultured in Dulbecco's modified eagles medium: F12 (1∶1) (Invitrogen) containing 10% (FBS), 100 U/ml penicillin, 100 µg/ml streptomycin and 2 mM L-glutamine (Sigma-Aldrich). Monolayer cultures were maintained in vented T75 cm^2^ flasks at 37°C, in a 5% CO_2_ (v/v) atmosphere. MG63 cells were cultured in Dulbecco's modified eagles medium (Invitrogen, Life Technologies) containing 10% (v/v) foetal bovine serum (FBS), 100 U/ml penicillin, 100 µg/ml streptomycin (Sigma-Aldrich) and 2 mM of L-glutamine (Sigma-Aldrich). Human articular chondrocytes (HACs) were isolated from articular cartilage obtained from patients with osteoarthritis undergoing total hip or knee replacement surgery. HACs were also obtained from non-OA patients who had undergone joint replacement due to neck-of-femur (NOF) fracture. Ethical approval and informed consent were obtained prior to surgery (research ethics committee reference 09/H0906/72 issued by the UK National Research Ethics Service). Enzymatic digestion and HAC culture was performed as previously described [55].

### Nucleic acid and protein extraction

Genomic DNA, total RNA and total protein were simultaneously extracted from SW872 cells using a spin column extraction kit according to the manufacturer's instructions (Nucleospin Triprep, Macherey-Nagel, supplied by Fisher, UK). Nucleic acids were quantified using a NanoDrop ND-1000 Spectrophotometer (NanoDrop Technologies, Wilmington, USA).

### Gene expression

1 µg of total RNA was DNase treated with 2 units of Turbo DNase (Ambion, Life Technologies) and reverse transcribed using Moloney Murine Leukemia Virus (M-MLV; Invitrogen) following manufacturer's instructions. Gene expression of *GDF5*, *SP1*, *SP3*, *P15* and *DEAF-1* was determined by real time RT-PCR and normalised to the housekeeping gene *HPRT1* using the delta ct method (2^−(ct^ ^test gene)-(ct HPRT1)^). Gene expression assays were purchased from either Applied Biosystems (ABI, Life Technologies) or Integrated DNA Technologies (IDT, Belgium). Differential Allelic Expression (DAE) analysis, to assess the expression of the C and T alleles of rs143383, was performed using a custom SNP genotyping assay (ABI, Life Technologies) containing forward and reverse primers and allele specific probes (VIC or FAM labelled). For analysis, the cDNA C/T allelic ratio was normalised to the genomic DNA (gDNA) C/T allelic ratio (representing a 1∶1 ratio) for each treatment group. An ABI PRISM 7900HT Sequence detection System was used for all real time PCR quantification. In SW872 cells for both overall gene expression and DAE analysis, three independent experiments were performed, with three biological replicates per experiment (n = 9). For each DNA and cDNA sample we performed three pipetting replicates, which were averaged prior to analysis. For SW1353 cells, three independent experiments were performed (n = 3). For HACs twelve biological replicates were performed. Statistical analysis of % knockdown was performed using a Students 2-tailed *t*-test whilst the one-way analysis of variance (ANOVA) test was used for *GDF5* fold change and DAE analysis. The primer and probe sequences are listed in Table S3A.

### Nuclear protein extraction

For the extraction of nuclear protein, cells were seeded at a density of 15×10^6^ on 500 cm^2^ plates (Corning, USA). Two buffers were used sequentially to isolate nuclear proteins; following centrifugation (10,000 g 30 seconds) cell pellets were re-suspended in 1 ml hypotonic buffer (10 mM HEPES pH 7.6, 1.5 mM MgCl_2_, 10 mM KCl, 1 mM DTT, 10 mM NaF, 1 mM Na_3_VO_4_, 0.1% Tergitol (v/v), 1× complete protease inhibitor cocktail tablet per 50 ml solution (Roche, UK)) and incubated on ice for 15 minutes. After a second centrifugation, the cell pellet was re-suspended in 500 µl high salt buffer (20 mM HEPES, pH 7.9, 420 mM NaCl, 20% glycerol (v/v), 1 mM DTT, 10 mM NaF, 1 mM Na_3_VO_4_, 1× complete protease inhibitor cocktail tablet per 50 mls of buffer) and incubated on ice for 30 minutes. Following a final centrifugation (10,000 g, 2 minutes), the supernatant containing nuclear protein was stored at −80°C.

### Bioinformatics search and Electrophoretic Mobility Shift Assays (EMSA)

PROMO 3.0, TESS, and TransFac online databases were used to predict protein binding to the C and T-alleles of *GDF5*. Fluorescently labelled oligonucleotides for both alleles (Eurofins MWG Operon, Ebersberg, Germany) were re-suspended to a final concentration of 100 pmol/µl in water (Sigma-Aldrich). Single-stranded oligonucleotides were incubated at 95°C for 5 minutes in a solution containing EMSA annealing buffer (100 mM Tris-HCl pH 7.5, 500 mM NaCl, 10 mM EDTA) to a final concentration of 20 pmol/µl and cooled slowly to room temperature for 2 hours to generate double stranded annealed probes. The annealed probes were diluted to 100 fmol/µl in water (Sigma-Aldrich) prior to the EMSA reaction. A native 5% (weight/volume) polyacrylamide gel was prepared the day before the EMSA and allowed to set at 4°C overnight. The EMSA was then carried out as per manufacturer's instructions using the Odyssey Infrared EMSA kit (LiCor Biosciences, Cambridge, UK). The optimal binding reaction contained 1× Binding Buffer, 2.5 mM DTT, 1 µg Poly (dI∶dC), 5 mM MgCl_2_, 200 fmol annealed oligonucleotide and 5 µg nuclear extract. The gel was visualised using an Odyssey Infrared Imager (LiCor Biosciences). For competition assays to test binding of predicted proteins, single stranded unlabelled oligonucleotides (Sigma-Aldrich) containing the consensus binding sequence of the protein were annealed as previously described for the labelled probes. For supershift EMSAs, 2 µg of antibody was added to the binding reaction. Table S1 lists the nucleotide sequences of the labelled probes and unlabelled competitor sequences. Table S4 provides details of the antibodies.

### Oligonucleotide pull down assay and quantitative tandem mass spectrometry

A 212 bp DNA region encompassing rs143383 was amplified by PCR using a biotinylated 5′ primer and unlabelled 3′ primer (Sigma-Aldrich) (Table S3B). Two PCRs were performed, using homozygous C or T template DNA at the polymorphic site. 40 pmol of PCR product was coupled to 2 mg of Streptavidin Dynabeads (Invitrogen) following the manufacturers instructions. A sample containing no DNA was used as a control. DNA-beads complexes were blocked as described previously [56]. SW872 cell nuclear lysates were extracted as described above, transferred to a tube for dialysis (Tube-O-dialyzer, VWR, UK), and dialyzed in a low salt buffer (20 mM HEPES pH 7.9, 20% (v/v) glycerol, 0.1 M KCl, 0.2 mM EDTA, 0.5 mM PMSF, 0.5 mM DTT) for 4 hours at 4°C. The buffer was replaced and the lysates were dialysed for a further 16 hours at 4°C. Following this, the nuclear lysate was pre-cleared for 1 hour with 50 µl Streptavidin Dynabeads (Invitrogen). DNA-bead complexes were then re-suspended in 1 mg of the prepared SW872 protein extract and incubated for 2 hours at 4°C with shaking. Beads were washed six times with BC-100 buffer and re-suspended in 1× SDS sample buffer (4% SDS, 0.2 M Tris-HCL, 4% glycerol, 0.01% bromophenol blue, 2% β-mercaptoethanol). Complexes were eluted from the beads following incubation at 95°C for 5 minutes and isolated following magnetic separation. The samples (CC, TT and no DNA) were loaded on to a 12% gel, and subject to separation by electrophoresis, followed by coomassie blue staining. Quantitative mass spectrometry was performed as previously described [57]. Briefly, following peptide digestion overnight using trypsin, labelling of the three conditions was carried out with a TMT isobaric mass tagging kit (Thermo Scientific, Surrey, UK). Labelled samples were mixed prior to off-gel fractionation of the peptides. Following liquid chromatography tandem mass spectrometry (LC-MS/MS), quantitative analysis was carried out using ProteinExplorer, version 1.0 (Thermo Scientific) and the search engine MASCOT (Matrix Science Company) used for identification of proteins. These results were then sorted according to detection in the background sample and ranked with the most robust hits being proteins with high confidence values, based on the identification of more than 2 unique peptide sequences, the coverage of peptides in the protein and those with low variability between peptide quantification values. Proteins known to have a role in transcriptional activation or repression were prioritised for further analysis.

### Chromatin Immunoprecipitation (ChIP)

ChIP experiments were performed as recommended by the manufacturer using the Magna ChIP A kit (Merck, Millipore, Consett, UK). Briefly, SW872 cells were cultured until 70% confluent on 500 cm^2^ culture plates (Corning). Cells were cross linked for 10 minutes with 1% (w/v) formaldehyde, 1.25 M glycine was then added for 5 minutes to quench unreacted formaldehyde. The cells were then washed twice and harvested in cold PBS containing protease inhibitors. Cells were then centrifuged for 8 minutes at 720 g, re-suspended in lysis buffer and incubated on ice for 20 minutes. The cell suspension was sonicated using a Soniprep150 probe sonicator (MSE UK, London, UK) to shear the chromatin, and then pre-cleared with magnetic protein A beads for 30 minutes at 4°C. 100 µg of chromatin was incubated with rotation overnight at 4°C in addition to 10 µg of either rabbit IgG antibody (negative control), anti-acetyl histone H3 (positive control) or 10 µg of the antibody of interest and 40 µl magnetic protein A beads (the antibodies used are listed in Table S4). Using a magnetic separator immunoprecipitated DNA/protein complexes were isolated and washed as recommended. Cross-linking was reversed by incubating the DNA/protein complexes and the input control (10% of sonicated chromatin) in elution buffer with proteinase K at 65°C for 2 hours. DNA was purified and analysed by PCR (Table S3B). 2 µl of immunoprecipitated DNA was added to a 15 µl PCR reaction, the thermocycling conditions as follows; 94°C 14 minutes, followed by 32 cycles of 94°C 30 seconds, 57°C for 30 seconds (annealing temperature for *GDF5* ChIP primers), 72°C for 30 seconds and a final step of 72°C for 5 minutes. PCR products were electrophoresed through a 2% (w/v) agarose gel containing ethidium bromide. Three ChIP experiments in total were performed for each antibody, each showing consistent results.

### RNA-mediated interference

SW872 cells were seeded at 350,000 cells per well in a 6 well culture plate (Costar, UK). After 24 hours, cells were transfected using 100 nM Dharmacon ON-TARGET*plus* Smartpool siRNAs targeted against *SP1*, *SP3*, *P15*, *DEAF-1* and a Non-Targeting Pool control in addition to Dharmafect 4 lipid reagent (Thermo Fisher, UK). After 48 hours the cells were harvested, nucleic acid and protein isolated and RNA reverse transcribed as described above. Depletion of mRNA expression was calculated compared to cells transfected with the ON-Target*plus* Non-Targeting Pool control siRNA (Thermo Fisher). SW1353 cells were seeded at 250,000 cells per well in a 6 well culture plate and transfected as described for SW872 cells using Dharmafect 1 lipid reagent (Thermo Fisher). Human articular chondrocytes were seeded at 300,000 cells per well in a 6 well culture plate and transfected as described for SW872 cells using Dharmafect 1 lipid reagent (Thermo Fisher).

### Reporter luciferase assays

The *GDF5* promoter and part of the 5′UTR region spanning −97 to +305 (relative to the transcriptional start site) was subcloned from the *GDF5* pGL3-Basic vector [46] into the *Mlu/BglII* sites of the purified pGL3-Enhancer Vector (Promega, UK). The Sp1, Sp3 and P15 open reading frames (ORF) were amplified from cDNA using the primers listed in Table S3B, ligated into the *EcoR1* and *SacII* sites of the pEGFP-N1 vector (Clontech) and transformed into MACH1 competent bacterial cells (Invitrogen). The DEAF-1-EGFP-N1 expression plasmid was kindly donated by C. Garrison Fathman [58]. Plasmid DNA was extracted using a Qiagen Maxiprep Kit (Qiagen, Crawley, UK). SW1353 cells were seeded at a density of 17,500 cells per well in a 48-well cell culture plate (Costar, UK) and cultured for 48 hours prior to transfection. Cells were transfected with 2 µg of plasmid DNA (containing 1 µg of *GDF5* pGL3 enhancer vector and several combinations of either 1 µg empty pEGFP-N1 vector, 500 ng empty pEGFP-N1 and one of the transcription factor expression plasmids, or 500 ng each of two transcription factor expression plasmids) in addition to 15 ng of pTK-RL Renilla using ExGen 500 *in vitro* transfection reagent (Fermentas, York, UK). Four wells were transfected per condition and a total of three individual experiments were performed. After 24 hours, transfected cells were lysed and luciferase and renilla activity measured using the Dual Luciferase Assay system (Promega, UK) with the MicroLumat Plus LB96V luminometer (Berthold Technologies UK, Harpenden, UK). Statistical analysis was performed using the Students 2-tailed *t*-test.

### Immunoblotting

To assess siRNA knockdown of our candidate proteins and successful over expression, total protein was isolated as described, quantified (Bradford reagent, Expedeon) and 10 µg was resolved on SDS-10% (w/v) polyacrylamide gels. Protein was then transferred to Immobilon-P PVDF membranes (Merck Millipore). The antibodies detailed in Table S4 were used to assess protein levels following siRNA knockdown and over expression in SW872 and SW1353 cells respectively. A monoclonal β-Actin antibody was used as a loading control. For the examination of protein over expression, 250,000 cells per well were seeded in 6-well culture dishes and transfected with plasmid vectors and ExGen500 as described for the 48-well plate, but the relative amounts of each were increased according to the culture volume. For the over expression of DEAF-1 EGFP, followed by DEAF-1 siRNA treatment, SW872 cells were transfected with DEAF-1 EGFP as described above, and after 6 hours the cells were treated with NTsiRNA or with DEAF-1 siRNA and then harvested after 48 hours.

### Immunofluorescence

To examine the overexpression of EGFP-N1 vectors, SW1353 cells were seeded at a density of 10,000 cells/well in a chamber slide (Nagel Nunc International, USA) and after 48 hours, transfected with 1 µg plasmid vector using ExGen 500. After 24 hours, cells were washed in PBS and fixed with 4% (w/v) Paraformaldehyde in PBS for 10 minutes, washed again in PBS and mounted using vectashield with DAPI (4′6-diamidino-2-phenylindole) (Vector Laboratories, Burlingame, CA). Fluorescence was detected using a LEICA DMLB fluorescent microscope and a SPOT-RT camera.

### Co-immunoprecipitation

SW1353 cells were cultured until 70% confluent on 500 cm^2^ culture plates (Corning) and nuclear protein was extracted as described above. 10 µg of antibody and 200 µg of nuclear extract (diluted 1 in 5 in high salt lysis buffer) was incubated over night at 4°C with shaking. 70 µl magnetic protein A beads were added to each immunoprecipitation, and this mixture was incubated at 4°C with shaking for 4 hours. Using a magnetic separator, immunoprecipitated protein complexes were isolated and washed with lysis buffer twice and with PBS once. The magnetic beads were then re-suspended in Laemmli buffer and the samples were heated to 95°C for 5 minutes. The supernatant was then taken forward for analysis by SDS-PAGE.

## Supporting Information

Figure S1 EMSA analysis of the binding region. The addition of increasing concentrations (10× and 50× the probe concentration) of the unlabelled competitors of varying sizes (full sized competitor, and three competitors covering different areas: Comp 1, Comp 2 and Comp 3) were added to the EMSA reactions containing the C or T allele probe. The sequences of each of the competitors are shown below the EMSAs, with the rs143383 polymorphism highlighted in bold and underlined.
(TIF)

Figure S2 Antibody supershift experiments performed on several additional *trans*-acting factors. Antibodies targeting E2F1 (E2F), EGR (EGR1), HDAC1 (H1), HDAC2 (H2), KLF16 (KLF) and Sp1 (positive control) were added to the EMSA reactions containing the C or T allele probe. Con, IgG rabbit antibody control.
(TIF)

Figure S3 EMSA analysis using different nuclear extracts. (**A**) Supershift experiment demonstrating the effect of adding antibodies targeting Sp1 and Sp3 to the EMSA reaction containing the T allele probe, compared to the IgG rabbit antibody control (Con). Nuclear extracts from SW872, SW1353 and MG63 cell lines and from human articular chondrocytes (HAC) were used. The arrows indicate the supershifted complexes. (**B**) Supershift experiment demonstrating the effect of adding antibodies targeting Sp1 and Sp3 to the EMSA reaction containing the C allele probe, compared to the IgG rabbit antibody control (Con). Nuclear extracts from SW872, SW1353 and MG63 cell lines and from human articular chondrocytes (HAC) were used. The arrows indicate the supershifted complexes. (**C**) Demonstration of the effect of adding P15 antibody to the EMSA reaction containing the C or T allele probe, compared to the IgG rabbit antibody control (Con). Nuclear extracts from SW872, SW1353 and MG63 cell lines and from human articular chondrocytes (HAC) were used. (**D**) Supershift experiment demonstrating the effect of adding an antibody targeting DEAF-1 to the EMSA reaction containing the C or T allele probe, compared to the IgG rabbit antibody control (Con). Nuclear extract from human articular chondrocytes (HAC) was used. The arrow indicates the supershifted complex.
(TIF)

Figure S4 EMSA analysis using alternative conditions. EMSA analysis demonstrating the effect of using both standard conditions and conditions to mimic the oligonucleotide pull down assay. All conditions contain either the C or the T allele probe and SW872 nuclear extract. Standard represents the normal EMSA conditions. Condition 1 is an EMSA reaction using the low salt oligonucleotide pull down buffer. Condition 2 represents 50% volume of the oligonucleotide pull down buffer diluted in water. Condition 3 represents the oligonucleotide pull down buffer in addition to 1 µg poly dI∶dC. Condition 4 represents the standard EMSA conditions in addition to 50 mM KCl, 2.5% glycerol and 0.1 mM EDTA to mimic those used in the pull down assay. The arrows highlight the Sp1 and Sp3 protein complexes.
(TIF)

Figure S5 Expression of *GDF5*, *Sp1*, *Sp3*, *P15* and *DEAF-1* in joint tissues. The expression levels of (**A**) *GDF5*, (**B**) *Sp1*, (**C**) *Sp3*, (**D**) *P15* and (**E**) *DEAF-1* were detected using real time PCR. The cartilage, synovium and fat pad tissue sample RNAs were extracted from OA patients following joint replacement surgery. NOF (neck of femur fracture) is RNA extracted from the cartilage taken from hip samples of patients without OA. Error bars denote the standard error of the mean (SEM). The data represents combined numbers of 30 OA cartilage, 12 NOF cartilage, 10 synovium and 10 fat pad samples.
(TIF)

Figure S6 Validation of DEAF-1 siRNA treatment. Examination of the effect of DEAF-1 siRNA treatment on DEAF-1 EGFP expression. Immunoblots demonstrating the effect of over expressing DEAF-1 EGFP (D1 EGFP) and the effect of concurrently depleting DEAF-1 expression using siRNA (DEAF-1 EGFP D1 siRNA). Protein extracted from cells that are over expressing DEAF-1 EGFP and that have been treated with the NTsiRNA control (D1 EGFP NTsiRNA) were used for assessing basal protein expression. β-Actin was used as a loading control. Arrow indicates DEAF-1 EGFP expression.
(TIF)

Figure S7 Knockdown of candidates in SW1353 chondrosarcoma cells and fold change in *GDF5* expression. (**A**) Expression levels of *Sp1*, *Sp3*, *P15* and *DEAF-1* mRNA are shown as a percentage of the control non-targeting siRNA (NTsiRNA) treated cells following *Sp1*, *Sp3*, *P15* and *DEAF-1* siRNA knockdown. Error bars denote the standard error of the mean (SEM). *p<0.05, ***p<0.001, calculated relative to the NTsiRNA value using a Students 2 tailed *t*-test. (**B**) Immunoblots demonstrating Sp1, Sp3, P15 and DEAF-1 protein depletion following siRNA treatment. Protein extracted from cells treated with the NTsiRNA control were used for basal protein expression whilst β-Actin was used as a loading control. (**C**) Fold change in *GDF5* expression following *Sp1*, *Sp3*, *P15* and *DEAF-1* siRNA knockdown and shown relative to the NTsiRNA control. Error bars denote the SEM. ***p<0.001, calculated using a ANOVA.
(TIF)

Figure S8 Knockdown of candidates in human articular chondrocytes. (**A**) Expression levels of *Sp1*, *Sp3*, *P15* and *DEAF-1* mRNA are shown as a percentage of the control non-targeting siRNA (NTsiRNA) treated cells following *Sp1*, *Sp3*, *P15* and *DEAF-1* siRNA knockdown. Error bars denote the standard error of the mean (SEM). *p<0.05, ***p<0.001, calculated relative to the NTsiRNA value using a Students 2 tailed *t*-test. (**B**) Immunoblots demonstrating Sp1, Sp3 and P15 protein depletion following siRNA treatment. Protein extracted from cells treated with the NTsiRNA control were used for basal protein expression whilst β-Actin was used as a loading control. (**C**) Fold change in *GDF5* expression following *Sp1*, *Sp3*, *P15* and *DEAF-1* siRNA knockdown in human articular chondrocytes relative to the NTsiRNA control. Error bars denote the SEM. *p<0.05, calculated using a ANOVA.
(TIF)

Figure S9 Immunofluorescence following over expression. Nuclei are stained blue with DAPI, shown in the first row. The localisation of the EGFP fusion proteins (Empty EGFP, Sp1 EGFP, Sp3 EGFP, P15 EGFP and DEAF-1 EGFP) is shown in the second row (EGFP). The final row shows the merged DAPI and EGFP images (Merge).
(TIF)

Figure S10 Co-immunoprecipitation (CoIP) of Sp1, Sp3, P15 and DEAF-1. Immunoprecipitations for Sp1, Sp3 and P15 were performed using SW1353 untransfected cell lysate, whilst DEAF-1 was immunoprecipitated with an EGFP antibody using SW1353 cell lysate over expressing DEAF-1 EGFP. Inputs represent 12.5% volume of untransfected lysate and 11.5% of transfected lysate. (**A**) Immunoblot examining the expression of Sp1. Immunoprecipitation with Sp1 antibody was used as a positive control, whilst a species matched IgG was used as a negative control. Sp3, P15 and DEAF-1 were immunoprecipitated to detect co-precipitating Sp1. The arrow highlights Sp1. (**B**) Immunoblot examining the expression of Sp3. Immunoprecipitation with Sp3 antibody was used as a positive control, whilst a species matched IgG was used as a negative control. Sp1, P15 and DEAF-1 were immunoprecipitated to detect co-precipitating Sp3. The arrows highlight Sp3. (**C**) Immunoblot examining the expression of P15. Immunoprecipitation with the P15 antibody was unsuccessful, thus could not be used as a positive control, whilst a species matched IgG was used as a negative control. Sp1, Sp3 and DEAF-1 were immunoprecipitated to detect co-precipitating P15. The arrow highlights P15. (**D**) Immunoblot examining the expression of DEAF-1. Immunoprecipitation of DEAF-1 using the EGFP antibody and the EGFP transfected lysate is shown. Sp1, Sp3 and P15 were immunoprecipitated using the untransfected lysate to detect co-precipitating endogenous DEAF-1. The arrow on the left highlights DEAF-1 EGFP and the arrow on the right highlights endogenous DEAF-1.
(TIF)

Table S1 The sequences of the rs143383 probes and of the competitor oligonucleotides used in the EMSA experiments. The forward primer sequences are shown. The consensus binding motif of the competitor proteins was identified using online prediction tools and is underlined. The flanking sequences were randomly generated.
(DOC)

Table S2 The C/T allelic ratios following over expression of the *trans*-acting factors. The promoter activities of the C and T *GDF5* luciferase vectors were compared to derive C/T ratios, which are shown for the *GDF5* vectors in addition to the empty EGFP vector (C/T) and for when these vectors were co-transfected in combination with Sp1 (C/T+Sp1), Sp3 (C/T+Sp3), P15 (C/T+P15), DEAF-1 (C/T+D1), Sp1 and Sp3 (C/T+Sp1+Sp3), Sp1 and DEAF-1 (C/T+Sp1+DEAF-1), and Sp3 and DEAF-1 (C/T+Sp3+DEAF-1). P-values were calculated using a Students 2 tailed *t*-test comparing the allelic ratios of each treatment group to either C/T, C/T+Sp1 (+Sp1), C/T+Sp3 (+Sp3) or C/T+D1 (+D1).
(DOC)

Table S3 The primers used in our experiments. (**A**) Nucleotide sequences of the primers and of the probes used for the real time RT-PCR assays measuring gene expression. (**B**) Nucleotide sequences of the primers used for creating the 212 bp fragment used in the oligonucleotide pull down assay, of the primers used for PCR following ChIP, and of the primers used to create the inserts for cloning in the overexpression vectors (the restriction enzyme sites used are underlined). F, Forward; R, Reverse.
(DOC)

Table S4 Details of the antibodies used in our experiments.
(DOC)
