# Supplementary figures and images for: The Identification of Trans-acting Factors That Regulate the Expression of GDF5 via the Osteoarthritis Susceptibility SNP rs143383
Source: PLoS Genet. 2013 Jun 27;9(6):e1003557. doi: 10.1371/journal.pgen.1003557 (PMC3694828; doi:10.1371/journal.pgen.1003557)

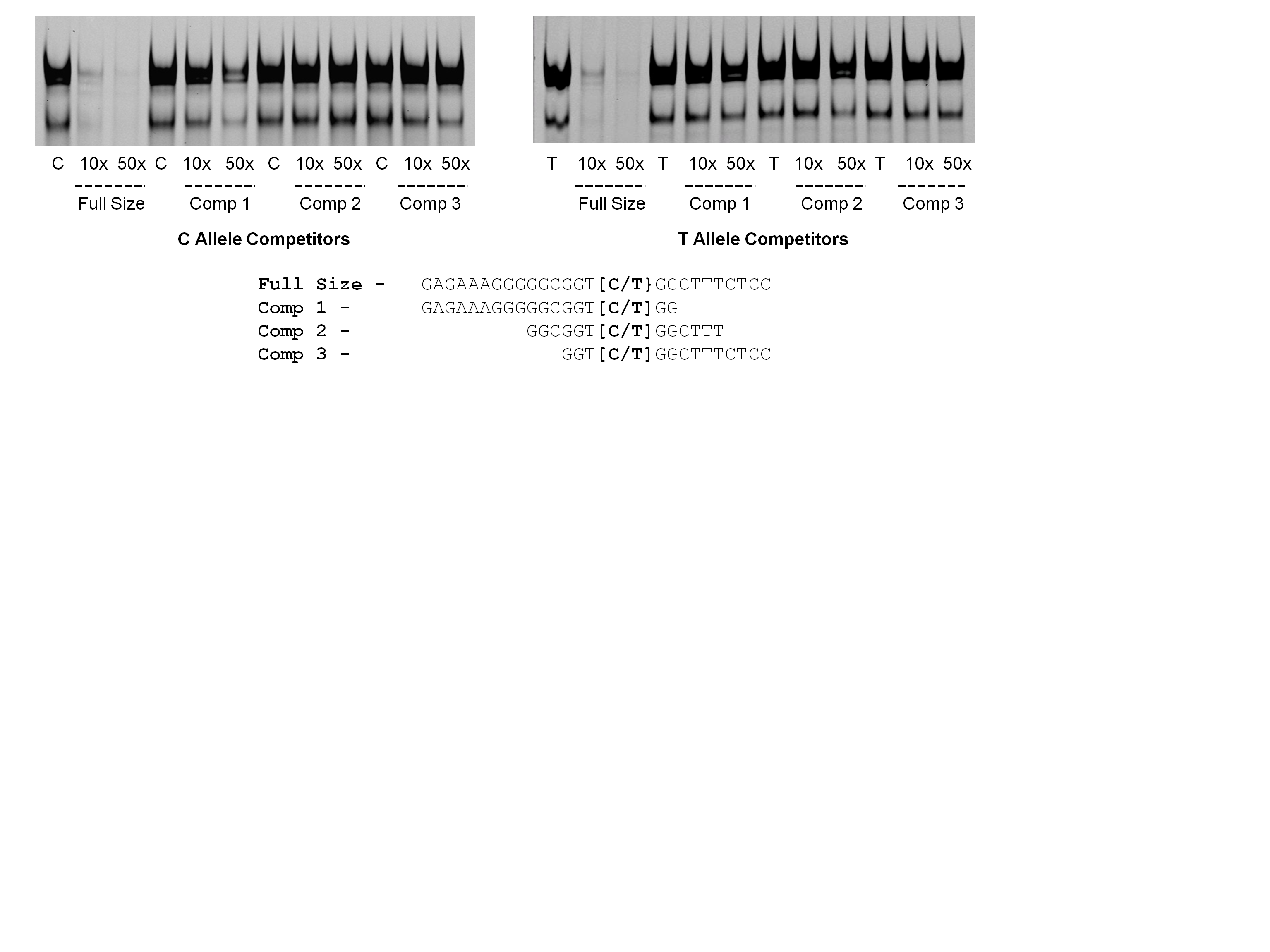

Supplement: Figure S1 — EMSA analysis of the binding region. The addition of increasing concentrations (10× and 50× the probe concentration) of the unlabelled competitors of varying sizes (full sized competitor, and three competitors covering different areas: Comp 1, Comp 2 and Comp 3) were added to the EMSA reactions containing the C or T allele probe. The sequences of each of the competitors are shown below the EMSAs, with the rs143383 polymorphism highlighted in bold and underlined. (TIF) [file pgen.1003557.s001.tif]

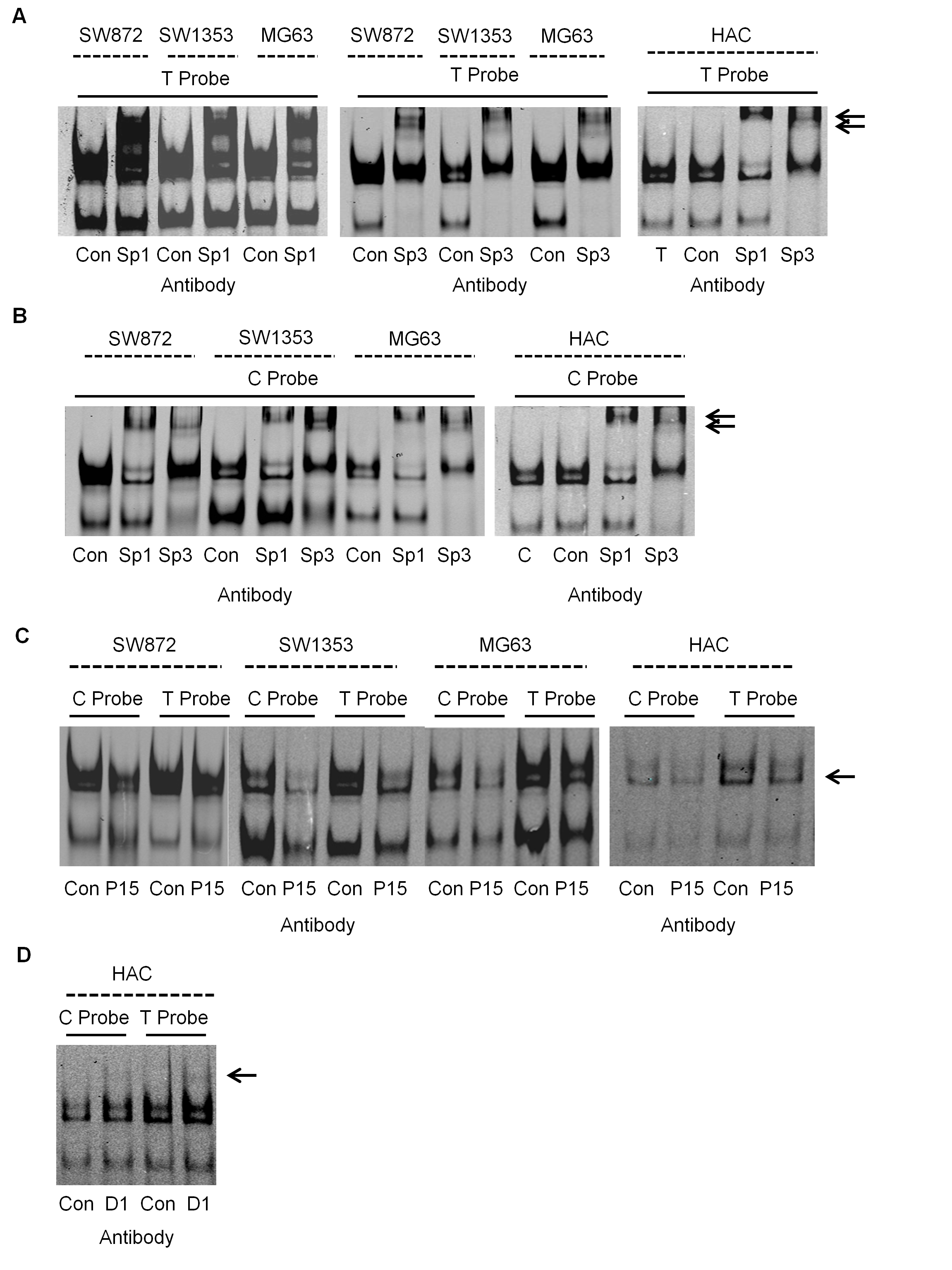

Supplement: Figure S3 — EMSA analysis using different nuclear extracts. (A) Supershift experiment demonstrating the effect of adding antibodies targeting Sp1 and Sp3 to the EMSA reaction containing the T allele probe, compared to the IgG rabbit antibody control (Con). Nuclear extracts from SW872, SW1353 and MG63 cell lines and from human articular chondrocytes (HAC) were used. The arrows indicate the supershifted complexes. (B) Supershift experiment demonstrating the effect of adding antibodies targeting Sp1 and Sp3 to the EMSA reaction containing the C allele probe, compared to the IgG rabbit antibody control (Con). Nuclear extracts from SW872, SW1353 and MG63 cell lines and from human articular chondrocytes (HAC) were used. The arrows indicate the supershifted complexes. (C) Demonstration of the effect of adding P15 antibody to the EMSA reaction containing the C or T allele probe, compared to the IgG rabbit antibody control (Con). Nuclear extracts from SW872, SW1353 and MG63 cell lines and from human articular chondrocytes (HAC) were used. (D) Supershift experiment demonstrating the effect of adding an antibody targeting DEAF-1 to the EMSA reaction containing the C or T allele probe, compared to the IgG rabbit antibody control (Con). Nuclear extract from human articular chondrocytes (HAC) was used. The arrow indicates the supershifted complex. (TIF) [file pgen.1003557.s003.tif]

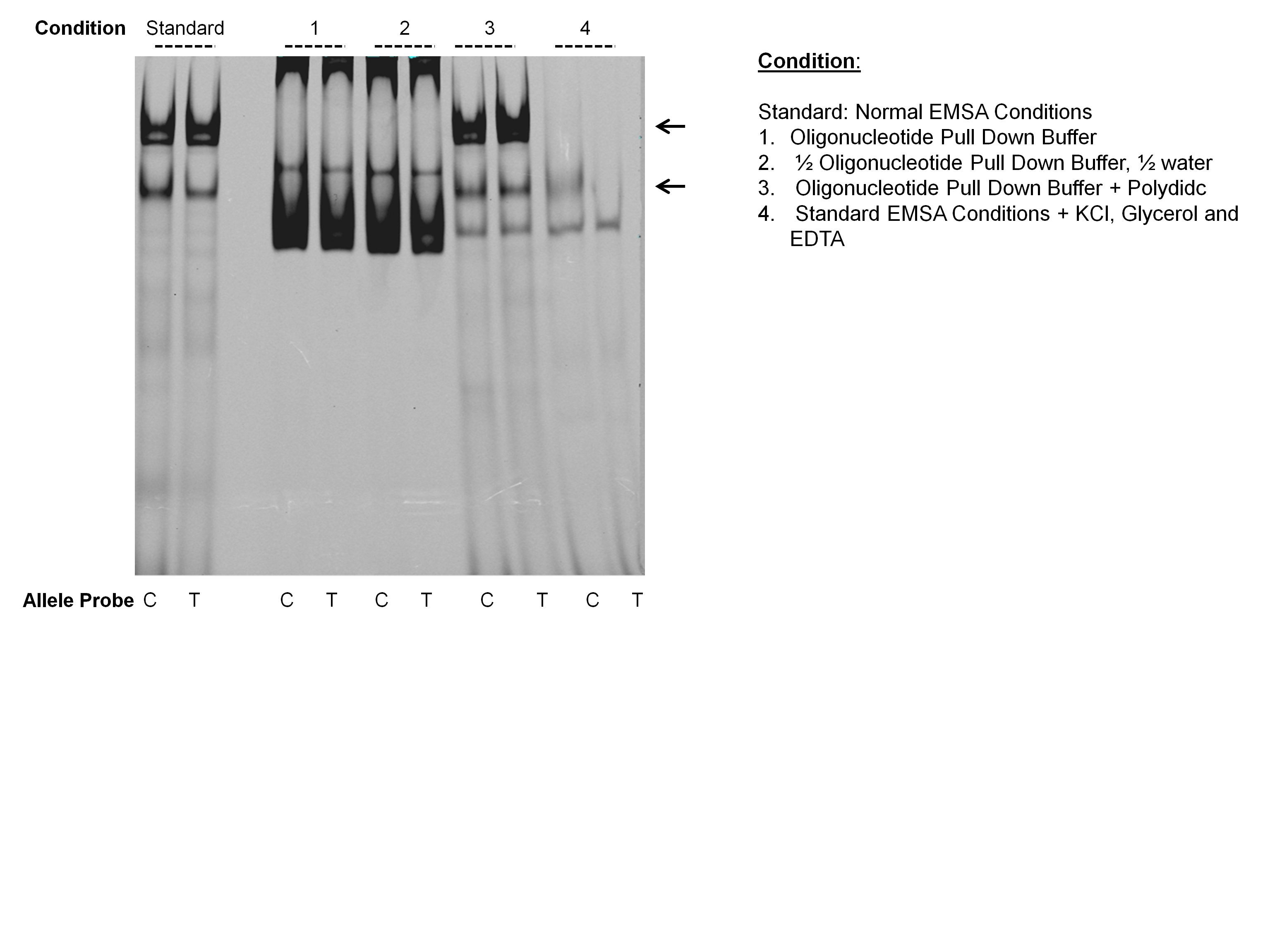

Supplement: Figure S4 — EMSA analysis using alternative conditions. EMSA analysis demonstrating the effect of using both standard conditions and conditions to mimic the oligonucleotide pull down assay. All conditions contain either the C or the T allele probe and SW872 nuclear extract. Standard represents the normal EMSA conditions. Condition 1 is an EMSA reaction using the low salt oligonucleotide pull down buffer. Condition 2 represents 50% volume of the oligonucleotide pull down buffer diluted in water. Condition 3 represents the oligonucleotide pull down buffer in addition to 1 µg poly dI∶dC. Condition 4 represents the standard EMSA conditions in addition to 50 mM KCl, 2.5% glycerol and 0.1 mM EDTA to mimic those used in the pull down assay. The arrows highlight the Sp1 and Sp3 protein complexes. (TIF) [file pgen.1003557.s004.tif]

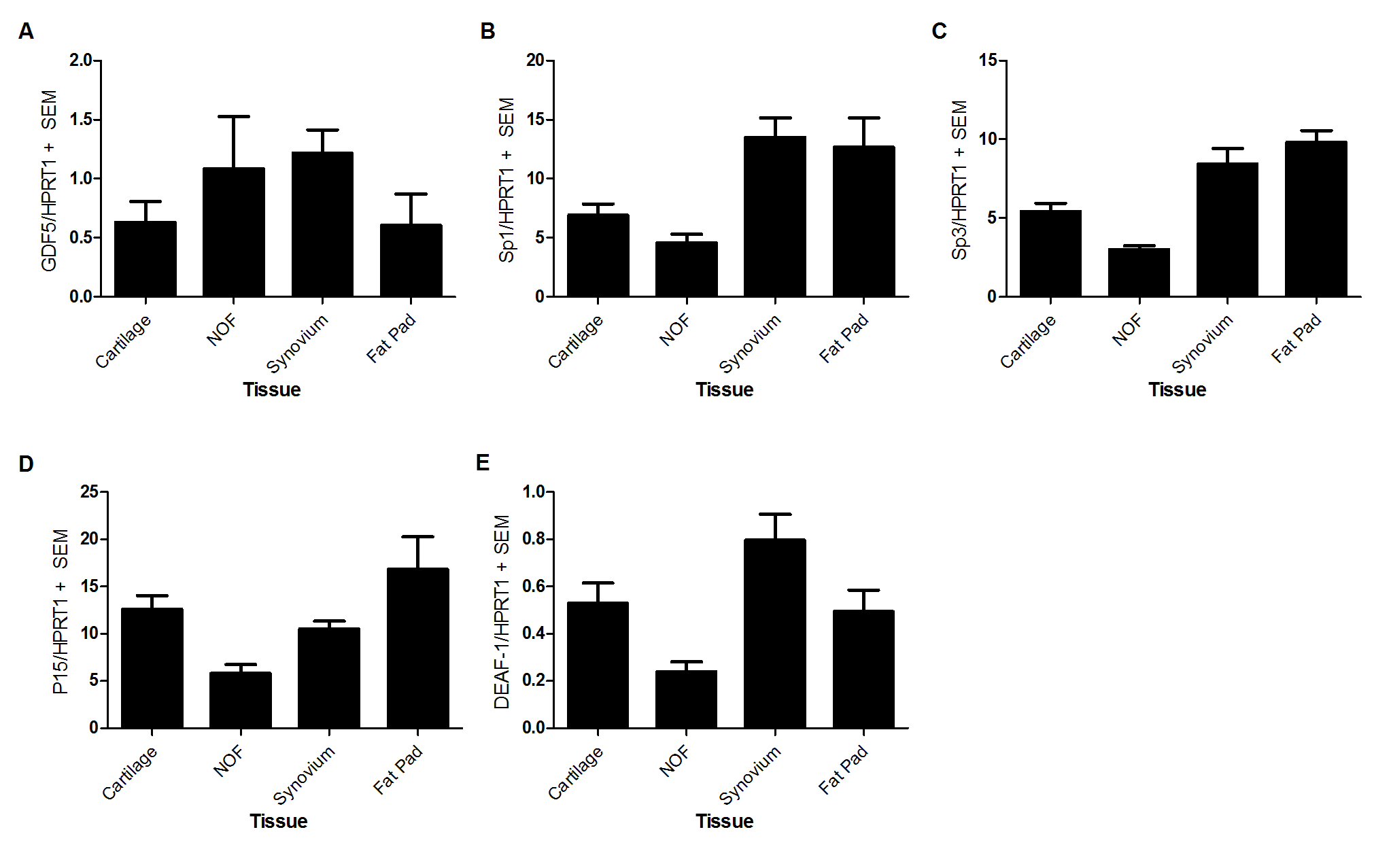

Supplement: Figure S5 — Expression of GDF5, Sp1, Sp3, P15 and DEAF-1 in joint tissues. The expression levels of (A) GDF5, (B) Sp1, (C) Sp3, (D) P15 and (E) DEAF-1 were detected using real time PCR. The cartilage, synovium and fat pad tissue sample RNAs were extracted from OA patients following joint replacement surgery. NOF (neck of femur fracture) is RNA extracted from the cartilage taken from hip samples of patients without OA. Error bars denote the standard error of the mean (SEM). The data represents combined numbers of 30 OA cartilage, 12 NOF cartilage, 10 synovium and 10 fat pad samples. (TIF) [file pgen.1003557.s005.tif]

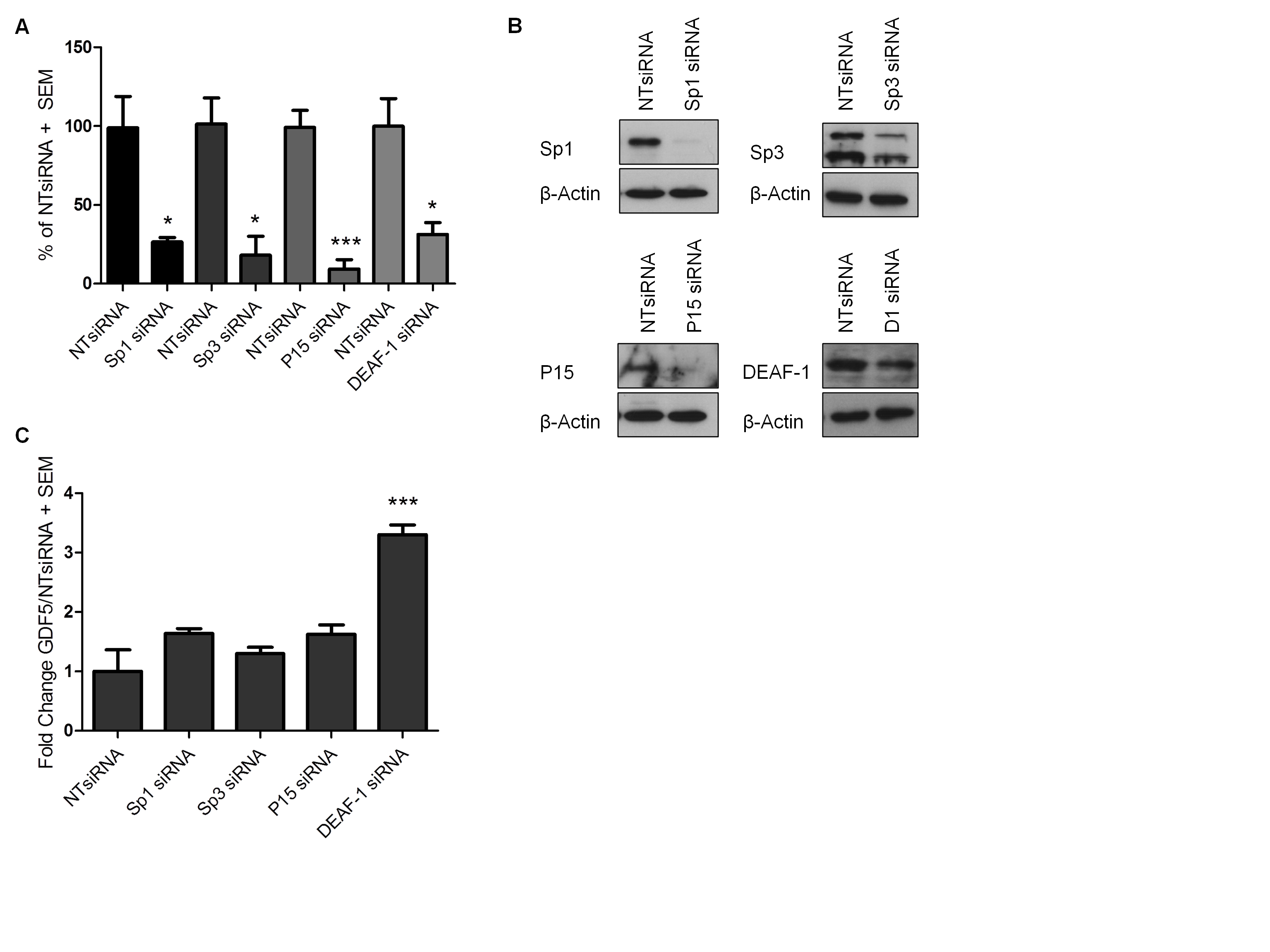

Supplement: Figure S7 — Knockdown of candidates in SW1353 chondrosarcoma cells and fold change in GDF5 expression. (A) Expression levels of Sp1, Sp3, P15 and DEAF-1 mRNA are shown as a percentage of the control non-targeting siRNA (NTsiRNA) treated cells following Sp1, Sp3, P15 and DEAF-1 siRNA knockdown. Error bars denote the standard error of the mean (SEM). *p<0.05, ***p<0.001, calculated relative to the NTsiRNA value using a Students 2 tailed t-test. (B) Immunoblots demonstrating Sp1, Sp3, P15 and DEAF-1 protein depletion following siRNA treatment. Protein extracted from cells treated with the NTsiRNA control were used for basal protein expression whilst β-Actin was used as a loading control. (C) Fold change in GDF5 expression following Sp1, Sp3, P15 and DEAF-1 siRNA knockdown and shown relative to the NTsiRNA control. Error bars denote the SEM. ***p<0.001, calculated using a ANOVA. (TIF) [file pgen.1003557.s007.tif]

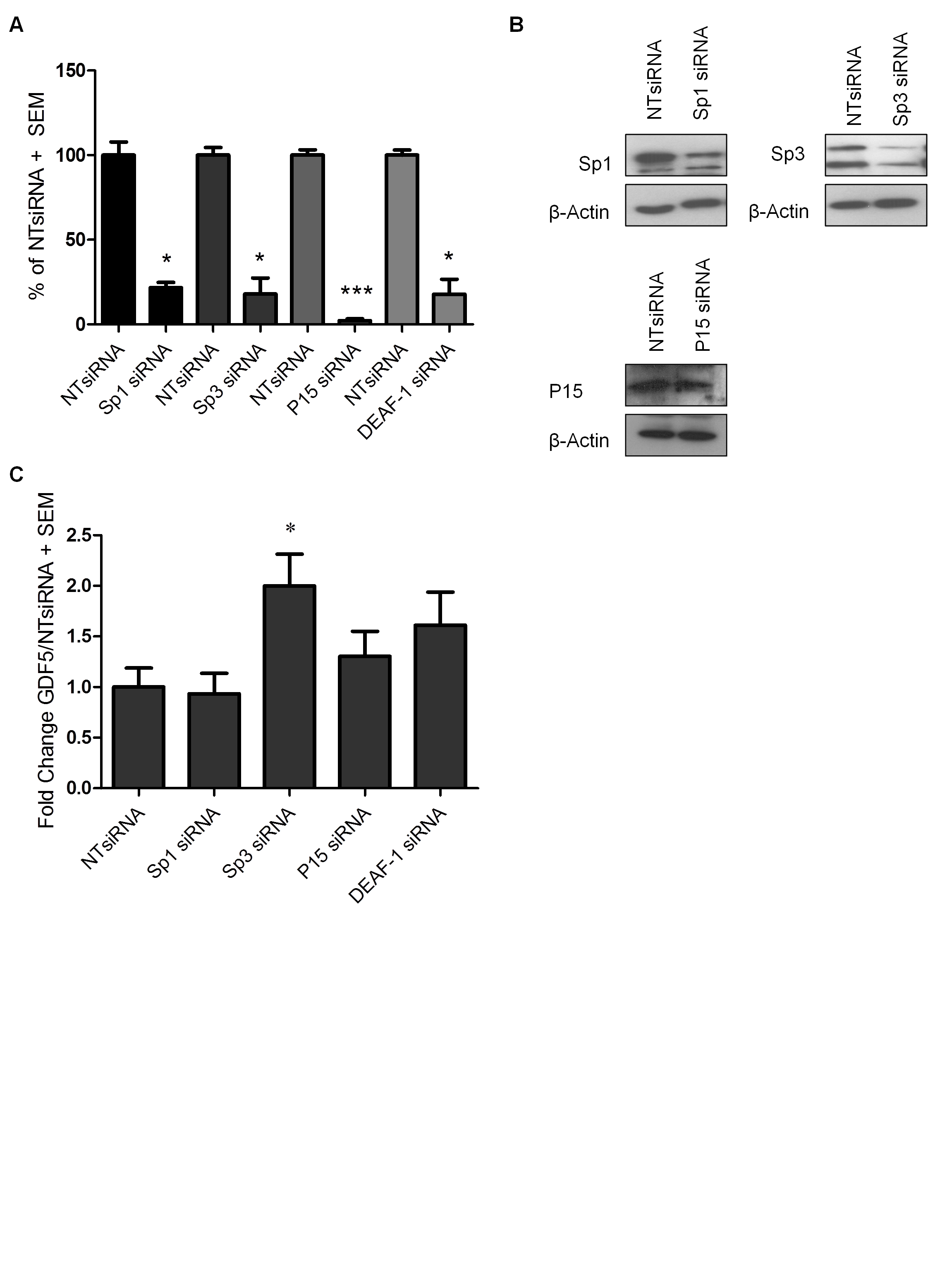

Supplement: Figure S8 — Knockdown of candidates in human articular chondrocytes. (A) Expression levels of Sp1, Sp3, P15 and DEAF-1 mRNA are shown as a percentage of the control non-targeting siRNA (NTsiRNA) treated cells following Sp1, Sp3, P15 and DEAF-1 siRNA knockdown. Error bars denote the standard error of the mean (SEM). *p<0.05, ***p<0.001, calculated relative to the NTsiRNA value using a Students 2 tailed t-test. (B) Immunoblots demonstrating Sp1, Sp3 and P15 protein depletion following siRNA treatment. Protein extracted from cells treated with the NTsiRNA control were used for basal protein expression whilst β-Actin was used as a loading control. (C) Fold change in GDF5 expression following Sp1, Sp3, P15 and DEAF-1 siRNA knockdown in human articular chondrocytes relative to the NTsiRNA control. Error bars denote the SEM. *p<0.05, calculated using a ANOVA. (TIF) [file pgen.1003557.s008.tif]

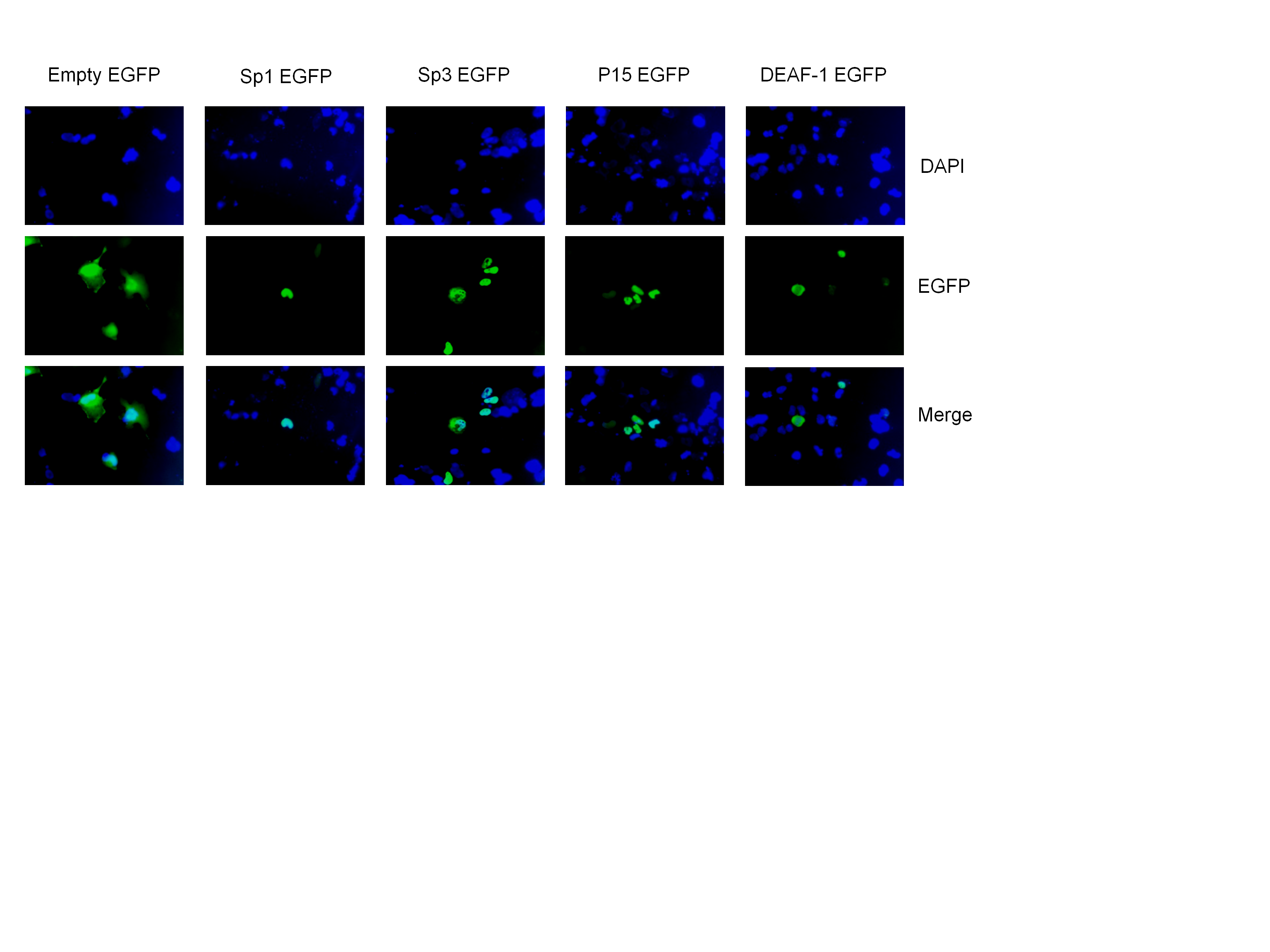

Supplement: Figure S9 — Immunofluorescence following over expression. Nuclei are stained blue with DAPI, shown in the first row. The localisation of the EGFP fusion proteins (Empty EGFP, Sp1 EGFP, Sp3 EGFP, P15 EGFP and DEAF-1 EGFP) is shown in the second row (EGFP). The final row shows the merged DAPI and EGFP images (Merge). (TIF) [file pgen.1003557.s009.tif]
