# Supplementary material for: The Identification of Trans-acting Factors That Regulate the Expression of GDF5 via the Osteoarthritis Susceptibility SNP rs143383
Source: PLoS Genet. 2013 Jun 27;9(6):e1003557. doi: 10.1371/journal.pgen.1003557 (PMC3694828; doi:10.1371/journal.pgen.1003557)
Supplement: Table S1 — The sequences of the rs143383 probes and of the competitor oligonucleotides used in the EMSA experiments. The forward primer sequences are shown. The consensus binding motif of the competitor proteins was identified using online prediction tools and is underlined. The flanking sequences were randomly generated. (DOC) [file pgen.1003557.s011.doc]

| Probe/Competitor | Sequence (5ʹ-3ʹ) |
| --- | --- |
| C allele labelled probe | GAGAAAGGGGGCGGTCGGCTTTCTCC |
| T allele labelled probe  Small Probe 1  Small Probe 2  Small Probe 3 | GAGAAAGGGGGCGGTTGGCTTTCTCC  GAGAAAGGGGGCGGTCGG  GGCGGTCGGCTTT  GGTCGGCTTTCTCC |
| Sp1/Sp3/ETF Competitor  cmyb Competitor | AATTGGGGGGGCGGGGGTACGTAGCA  AATTGGACCGGCGGTTGTACGTAGCA |
| E2F1 Competitor | AATTGGACCAGGGCGGGTACGTAGCA |
| EllaEa Competitor | AATTGGGAGGGCGTTAGTACGTAGCA |
| EGR1 Competitor | AATTGTGTGGGCGGGAGCACGTAGCA |
| GCF Competitor | AATTGGAAGCGCGGGCCGACGTAGCA |
| UHRF1Competitor  RC2 Competitor  IA1 Competitor  P53 Competitor  NF1 Competitor  GABP Competitor  CP2 Competitor  CTF Competitor  DRF1.1 Competitor  KLF16 Competitor | AATTGGACCTGGCATTGGACGTAGCA  AATTGGACCTGGGGTTTAACGTAGCA  ATGTAAGGGGGCGATAGTACGTAGCA  AATTGGACCTGGGCATGTACGTAGCA  AATTGGACCTGGCTTTGGCCGTAGCA  AATTGGACAACCCCCCGTACGTAGCA  AATTGGACCTGGCCCAATACGTAGCA  AATTGGACCTGCGTTTGGACGTAGCA  AATTGGACCGGCGGTGACTCGTAGCA  AATTGGAGGGGCGGTGGTACGTAGCA |
