# Supplementary material for: The Identification of Trans-acting Factors That Regulate the Expression of GDF5 via the Osteoarthritis Susceptibility SNP rs143383
Source: PLoS Genet. 2013 Jun 27;9(6):e1003557. doi: 10.1371/journal.pgen.1003557 (PMC3694828; doi:10.1371/journal.pgen.1003557)
Supplement: Table S2 — The C/T allelic ratios following over expression of the trans-acting factors. The promoter activities of the C and T GDF5 luciferase vectors were compared to derive C/T ratios, which are shown for the GDF5 vectors in addition to the empty EGFP vector (C/T) and for when these vectors were co-transfected in combination with Sp1 (C/T+Sp1), Sp3 (C/T+Sp3), P15 (C/T+P15), DEAF-1 (C/T+D1), Sp1 and Sp3 (C/T+Sp1+Sp3), Sp1 and DEAF-1 (C/T+Sp1+DEAF-1), and Sp3 and DEAF-1 (C/T+Sp3+DEAF-1). P-values were calculated using a Students 2 tailed t-test comparing the allelic ratios of each treatment group to either C/T, C/T+Sp1 (+Sp1), C/T+Sp3 (+Sp3) or C/T+D1 (+D1). (DOC) [file pgen.1003557.s012.doc]

| **Vector** | **C/T Ratio** | **P Value** |
| --- | --- | --- |
| **C/T**  **C/T + Sp1**  **C/T + Sp3**  **C/T + P15**  **C/T + D1**  **C/T + Sp1 + Sp3**  **C/T + Sp1 + D1**  **C/T + Sp3 + D1** | 1.2  1.38  1.48  1.24  1.37  1.70  1.55  1.6 | 0.005  <0.0001  0.56  0.006  <0.0001 (vs. C/T)  < 0.001 (vs. + Sp1)  0.03 (vs. + Sp3)  <0.0001 (vs. C/T)  0.1 (vs. + Sp1)  0.1 (vs. + D1)  <0.0001 (vs. C/T)  0.2 (vs. + Sp3)  0.01 (vs. + D1) |
