# Supplementary material for: The Identification of Trans-acting Factors That Regulate the Expression of GDF5 via the Osteoarthritis Susceptibility SNP rs143383
Source: PLoS Genet. 2013 Jun 27;9(6):e1003557. doi: 10.1371/journal.pgen.1003557 (PMC3694828; doi:10.1371/journal.pgen.1003557)
Supplement: Table S3 — The primers used in our experiments. (A) Nucleotide sequences of the primers and of the probes used for the real time RT-PCR assays measuring gene expression. (B) Nucleotide sequences of the primers used for creating the 212 bp fragment used in the oligonucleotide pull down assay, of the primers used for PCR following ChIP, and of the primers used to create the inserts for cloning in the overexpression vectors (the restriction enzyme sites used are underlined). F, Forward; R, Reverse. (DOC) [file pgen.1003557.s013.doc]

**A**

| **Gene** | **Primers (5ʹ to 3ʹ)** | **Probe (5ʹ to 3ʹ)** |
| --- | --- | --- |
| *HPRT1* | F: TGCTGAGGATTTGGAAAGGG  R: ACAGAGGGCTACAATGTGATG | AGGACTGAACGTCTTGCTCGAGATG |
| *GDF5* | ABi Assay ID: Hs00167060_m1, part number: 4448892 | |
| *Sp1* | F: TCAACTCTCCTCCATGCCA  R: CAGGTGATCATGGAGCTCAG | ACCTGGATTCCTGAAGTACCCAATGC |
| *Sp3* | F: AGTTAGTCTAAGCACTGGTCAG  R: GAAGAACCTGATCCTGAAGAGTG | ATCTGCAGGACTGTCAGCATTCTCTC |
| *P15* | F: GAAGCGATGCCTAAATCAAAGG  R: AGACAGGTGAGACTTCGAGAG | CAACCTCACTGTCAGAATCACTGCCA |
| *DEAF-1* | F: GTACAGTCCCACCGAGTTTG  R: GGATCTTAAACCCTCACGCT | ACCCTTGCAGTGCCTC |
| *GDF5* DAE | F: AGTCAGTTGTGCAGGAGAAAGG  R: TTCAAGAACGAGTTATTTTCAGCTGC | GGCGGTTGGCTTTCT (VIC) / GGCGGTCGGCTTTCT (FAM) |

**B**

| **Primer Name** | **Primers (5ʹ to 3ʹ)** |
| --- | --- |
| Oligonucleotide Pull Down *GDF5* | F: [biotin]CGTCGAATTCGCATTACGCCATTCTTCCTTC  R: CGGGTGTGTGTTTGTATCCAG |
| ChIP *GDF5* (Exon 1) | F: CTTCAAGCCCTCAGTCAGTTG  R: CTGGATACAAACACACACCCG |
| *Sp1* (*EcoR1)/(SacII)* | F: 5ʹ-GGGGGAATTCATGGATGAAATGACAGCTGTG-3ʹ  R: 5ʹ-GGGGCCGCGGGAAGCCATTGCCACTGATATT-3ʹ |
| *Sp3* (*EcoR1)/(SacII)* | F: GGGGGAATTCATGACCGCTCCCGAAAAGCCC  R: GGGGCCGCGGCTCCATTGTCTCATTTCCAGA |
| *P15 (EcoR1)/(SacII)* | F: GGGGGAATTCATGCCTAAATCAAAGGAACT  R: GGGGCCGCGGCAGTTTTCTTACTGCATCATC |
