# Supplementary material for: The Identification of Trans-acting Factors That Regulate the Expression of GDF5 via the Osteoarthritis Susceptibility SNP rs143383
Source: PLoS Genet. 2013 Jun 27;9(6):e1003557. doi: 10.1371/journal.pgen.1003557 (PMC3694828; doi:10.1371/journal.pgen.1003557)
Supplement: Table S4 — Details of the antibodies used in our experiments. (DOC) [file pgen.1003557.s014.doc]

| **Protein targeted by the antibody** | **Company from where the antibody was purchased** | **Catalogue number at the company for the antibody** | **Species in which the antibody was generated** | **Dilution used for immunoblotting** |
| --- | --- | --- | --- | --- |
| DEAF-1 | Donation from the group of Paul Albert [44] | n/a | Rabbit | 1:1000 |
| β-Actin | Sigma-Aldrich | A5316 | Mouse | 1:2000 |
| IgG from rabbit serum | Sigma-Aldrich | I5006 | Rabbit | n/a |
| Sp1 | Santa-cruz | Sc-59 (PEP2) | Rabbit | 1:200 |
| Sp3 | Santa-cruz | Sc-644 (D-20) | Rabbit | 1:200 |
| P15 | Santa-cruz | Sc-48778 (H-114) | Rabbit | 1:200 |
| E2F1 | Santa-cruz | Sc193 (C-20) | Rabbit | n/a |
| HDAC1 | Abcam | Ab7028 | Rabbit | n/a |
| HDAC2 | Abcam | Ab7029 | Rabbit | n/a |
| EGR-1 | Santa-cruz | Sc-20689 (H-250) | Rabbit | n/a |
| KLF16 | Santa-cruz | Sc-131168 | Rabbit | n/a |
